# Sarcopenia as a risk factor for nonalcoholic fatty liver disease and liver fibrosis: an updated systematic review and meta-analysis

**DOI:** 10.3389/fnut.2026.1726600

**Published:** 2026-03-09

**Authors:** Xinrong Zuo, Doudou Chen, Yi Kang, Xinyi Hu, Yarong Zhang, Kuo Tang, Junhua Shi, Tao Li, Wangning Shangguan

**Affiliations:** 1 Department of Anesthesiology and Perioperative Medicine, The Second Affiliated Hospital and Yuying Children's Hospital of Wenzhou Medical University, Wenzhou, China; 2 Department of Anesthesiology, Laboratory of Mitochondrial Metabolism and Perioperative Medicine, National-Local Joint Engineering Research Centre of Translational Medicine of Anesthesiology, West China Hospital of Sichuan University, Chengdu, Sichuan, China; 3 Metabolomics and Proteomics Technology Platform, West China Hospital, Sichuan University, Chengdu, Sichuan, China; 4 State Key Laboratory of Oral Diseases & National Center for Stomatology & National Clinical Research Center for Oral Diseases, West China Hospital of Stomatology, Sichuan University, Chengdu, Sichuan, China

**Keywords:** hepatic fibrosis, metabolic dysfunction-associated steatotic liver disease, nonalcoholic fatty liver disease, prevalence, sarcopenia

## Abstract

**Background:**

Sarcopenia, an age-related skeletal muscle disorder with metabolic dysregulation, has been linked to various chronic diseases. Emerging evidence suggests a bidirectional relationship with nonalcoholic fatty liver disease (NAFLD), though the epidemiological and mechanistic links remain unclear. This study aimed to assess the prevalence of sarcopenia in NAFLD populations and its association with NAFLD and liver fibrosis.

**Methods:**

PubMed, EMBASE, MEDLINE, Cochrane Library, and Web of Science databases were searched for eligible studies published until August 6, 2025. Two validated tools assessed the study quality and risk of bias. Statistical analyses were conducted using STATA 17.0. This study has been registered on PROSPERO (CRD420251021280).

**Results:**

From 4,938 screened records, 41 studies (*n* = 185,575) were retrieved. The prevalence of sarcopenia in patients with NAFLD varied substantially (0.8 to 80.9%), with a pooled estimate significantly higher than that in non-NAFLD controls 23% (95% CI: 20 to 26%) vs. 15% (95% CI: 13 to 17%). Meta-analysis revealed that sarcopenia was associated with a 1.58-fold increased risk of NAFLD (95% CI: 1.37 to 1.82) and a 2.03-fold risk of concomitant liver fibrosis (95% CI: 1.54 to 2.68). Subgroup analyses showed that the association was consistent across different muscle mass assessment methods, but varied by NAFLD diagnostic modality. CT-based NAFLD diagnosis yielded the highest aORs, while transient elastography showed a non-significant association.

**Conclusion:**

This study confirms a high prevalence of sarcopenia in NAFLD populations and a significant association with NAFLD and liver fibrosis, supporting its role as a modifiable risk factor. However, heterogeneity due to diagnostic variability remains a limitation. Standardized criteria and longitudinal studies are needed to establish causality.

**Systematic review registration:**

https://www.crd.york.ac.uk/PROSPERO/, PROSPERO: CRD420251021280.

## 1 Introduction

Sarcopenia is a geriatric syndrome characterized by progressive decline in muscle strength, mass, and physical performance. While primary sarcopenia is mainly age-related, secondary sarcopenia is linked to chronic conditions such as cardiopulmonary, hepatic, renal, metabolic, and neurological diseases (1, 2). Its pathogenesis involves chronic inflammation, inactivity, malnutrition, hormonal dysregulation, gut microbiota imbalance, and metabolic disturbances (3). Since its introduction in 1989, diagnostic criteria have evolved, shifting focus from low muscle mass to muscle weakness as the key indicator. However, variability in assessment methods leads to widely differing prevalence estimates (5 to 22%) (1, 4). Currently, no approved pharmacological treatments exist beyond exercise and nutritional interventions, although myostatin inhibitors and hormonal therapies have shown potential (4). Sarcopenia increases the risk of fractures, disability, and the healthcare burden, making it a growing global public health challenge.

Nonalcoholic fatty liver disease (NAFLD) is the most prevalent liver disorder worldwide, encompassing a disease spectrum ranging from simple steatosis to the more aggressive inflammatory phenotype of nonalcoholic steatohepatitis. As the disease progresses, it progresses to fibrosis, cirrhosis, and hepatocellular carcinoma, driving significant liver-related mortality (5, 6). Its global prevalence is 32.4% in adults, with 46.9 new cases per 1,000 person-years, disproportionately affecting obese populations (75%) owing to its strong ties to insulin resistance and type 2 diabetes (7, 8). Recent mechanistic insights have prompted a redefinition of metabolic dysfunction-associated steatotic liver disease (MASLD), emphasizing metabolic dysregulation as the core pathogenic driver (9). While MASLD reframes the diagnosis around positive cardiometabolic criteria rather than exclusion of other causes, we retain the term NAFLD throughout this review to align with the diagnostic definitions used in most included studies. Often asymptomatic yet linked to hepatic and systemic complications and with no approved drugs, NAFLD remains a critical public health threat (6, 7, 9).

Sarcopenia and NAFLD are two major global health burdens sharing overlapping mechanisms, including insulin resistance, chronic inflammation, endocrine dysfunction, gut dysbiosis, and metabolic/nutritional imbalances (10, 11). Bidirectional crosstalk along the liver-muscle axis creates a pathological feedback loop. Hepatic steatosis and fibrosis drive proinflammatory signaling, worsening systemic insulin resistance. This further aggravates myosteatosis and muscle loss, impairing hepatic perfusion and amplifying lipotoxicity via myokine-mediated pathways (10, 11). Clinically, NAFLD patients exhibit reduced muscle strength and mass, while sarcopenia accelerates NAFLD progression (12, 13). However, reported sarcopenia prevalence in NAFLD varies widely (0.8 to 80.9%), reflecting differences in diagnostic criteria (EWGSOP, AWGS, FNIH), populations, and NAFLD assessment methods (14, 15).

Previous meta-analyses examined the sarcopenia-NAFLD association but had significant limitations: overlapping study populations biasing prevalence estimates, and problematic pooling of bidirectional effect measures. In particular, combining aORs from studies with sarcopenia as outcome with those examining NAFLD obscures causal interpretation. To address this, we conducted an updated systematic review with three objectives: to provide reliable estimates of sarcopenia prevalence in NAFLD populations, to assess the association between sarcopenia and the incidence of NAFLD, and to examine its association with progressive hepatic fibrosis. This study adheres to TITAN Guidelines 2025 and no artificial intelligence tools were used.

## 2 Methods

### 2.1 Databases and search strategy

This study protocol was registered in PROSPERO and follows PRISMA (16) (Preferred Reporting Items for Systematic Reviews and Meta-Analyses) and AMSTAR 2 (17) (Assessing the Methodological Quality of Systematic Reviews) guidelines. Two independent reviewers comprehensively searched all relevant articles published up to August 6, 2025, on PubMed, Embase, Medline, Cochrane Library, and Web of Science databases, and manually screened the citations included in these articles. The detailed search strategies are shown in Supplementary Table S1. No language or publication-type restrictions were imposed.

### 2.2 Inclusion and exclusion criteria

All included studies met the following criteria: (1) cross-sectional or cohort studies; (2) population: ≥18 years old, patients with NAFLD/MASLD hospitalized or diagnosed according to clear diagnostic criteria; (3) sarcopenia defined as comprehensive criteria incorporating low muscle mass (LMM), low muscle strength (LMS), low physical performance (LPP), or non-comprehensive criteria evaluating at least one of these components; (4) lean skeletal muscle mass detected using instruments commonly used in previous studies, such as dual-energy X-ray absorptiometry (DXA), bioelectrical impedance analysis (BIA), magnetic resonance imaging (MRI), and computed tomography (CT); and (5) provision of either (a) quantitative prevalence estimates of sarcopenia in NAFLD populations or (b) aORs with corresponding 95% confidence intervals (CI) evaluating NAFLD/liver fibrosis risk in sarcopenic individuals.

Studies were excluded based on the following criteria: (1) failure to report the primary outcomes of interest (sarcopenia prevalence or NAFLD/liver fibrosis risk estimates), (2) enrolled non-NAFLD/MASLD, and (3) non-original research, such as reviews, case reports, editorials, letters, comments, conference abstracts, and animal studies.

### 2.3 Study selection

Following automated deduplication using EndNote, two independent reviewers conducted a two-phase screening process. First, they evaluated the titles/abstracts and then performed full-text assessments using the predefined PRISMA-compliant criteria. For overlapping cohorts, we prioritized studies with larger sample sizes or more recent publication dates. Discrepancies were resolved by consensus with a third reviewer.

### 2.4 Data extraction

Using standardized forms (Tables 1, 2), we extracted the following: (1) sarcopenia prevalence data (including diagnostic methods such as the SARC-F questionnaire (a simple five-item questionnaire), cut-off values, and criteria) from NAFLD and non-NAFLD populations, and (2) aORs for NAFLD/liver fibrosis risk in sarcopenia patients. The extracted variables included study characteristics (author, year, region, and design), population demographics (sample size, age, sex distribution, and BMI), and assessment methods for both sarcopenia and NAFLD/fibrosis. The covariate adjustment details for aORs are presented in Supplementary Table S2. Our primary outcomes were the pooled sarcopenia prevalence in NAFLD and the association between sarcopenia and NAFLD/fibrosis risk (expressed as aORs with 95% CI).

**Table 1:** Characteristics of the included studies and main outcomes of sarcopenia and NAFLD.

First author and year	NAFLD	Study region	Study design	Sample size	Number of NAFLD	Male, *n* (%)	Age^a^ (years)	BMI^a^ (kg/m^2^)	Prevalence of sarcopenia *n* (%)	Criteria (assessment method to detect sarcopenia)	Muscle mass measure	Cut-off value for muscle mass	Sarcopenia diagnostic criteria	NAFLD diagnosis
Bhanji (12)	NAFLD (+)	North America	cohort	265	136	70 (52.0)	60.2	34.9	30 (22.1)	LMM (CT)	CT	SMI < 50 cm^2^/m^2^ in men and < 39 cm^2^/m^2^ in women	Carey EJ (90)	NA
Linge (15)	NAFLD (+)	Europe	cross-sectional	5,326	1,204	644 (53.5)	62.9	30.1	19 (1.6)	LMM (DXA) + LMS (HGS)	DXA	ASM/ht^2^: <7.0 kg/m^2^ in men and <6.0 kg/m^2^ in women	EWGSOP2 (2019)	MRI
Linge (15)	NAFLD (−)	Europe	cross-sectional	5,326	4,122	1702 (41.3)	62.6	25.4	142 (3.4)	LMM (DXA) + LMS (HGS)	DXA	ASM/ht^2^: <7.0 kg/m^2^ in men and <6.0 kg/m^2^ in women	EWGSOP2 (2019)	MRI
Guan (45)	NAFLD (+)	Asia	cross-sectional	1,363	354	NA	NA	NA	55 (15.5)	LMM (BIA) + LMS (HGS)/LPP (6 m GS)	BIA	ASM/ht^2^: <7.0 kg/m^2^ in men and <5.7 kg/m^2^ in women	AWGS (2019)	Abdominal US
Guan (45)	NAFLD (−)	Asia	cross-sectional	1,363	1,009	NA	NA	NA	31 (2.8)	LMM (BIA) + LMS (HGS)/LPP (6 m GS)	BIA	ASM/ht^2^: <7.0 kg/m^2^ in men and <5.7 kg/m^2^ in women	AWGS (2019)	Abdominal US
Kang (44)	NAFLD (+)	Asia	cross-sectional	683	683	588 (86.1)	49.4	26.4	75 (11.0)	LMM (BIA)	BIA	ASM/BMI < 0.789 in men or <0.512 in women	FNIH	Abdominal US
Debroy (37)	NAFLD (+)	Europe	cross-sectional	169	57	57 (100)	56.5	25.5	10 (20.8)	LMM (DXA)	DXA	ASM/ht^2^ < 7.26 kg/m^2^	Baumgartner (91)	CT
Debroy (37)	NAFLD (−)	Europe	cross-sectional	169	112	112 (100)	57	24.1	32 (31.1)	LMM (DXA)	DXA	ASM/ht^2^ < 7.26 kg/m^2^	Baumgartner (91)	CT
Moon (50)	NAFLD (+)	Asia	cross-sectional	28,060	6,488	3,380 (52.1)	50.1	27.6	1,019 (15.7)	LMM (DXA)	DXA	ASM/BMI < 0.789 in men or <0.512 in women	FNIH	HSI value
Moon (50)	NAFLD (−)	Asia	cross-sectional	28,060	21,572	10,117 (46.9)	50.7	22.9	1,510 (7.0)	LMM (DXA)	DXA	ASM/BMI < 0.789 in men or <0.512 in women	FNIH	HSI value
Nachit (47)	NAFLD (+)	Europe	cohort	184	150	50 (33.3)	41	40.6	2 (1.3)	LMM (CT)	CT	SMI < 50 cm^2^/m^2^ in men and < 39 cm^2^/m^2^ in women	Carey EJ (90)	Liver biopsy
Nachit (47)	NAFLD (−)	Europe	cohort	184	34	4 (11.8)	40	38.3	6 (17.6)	LMM (CT)	CT	SMI < 50 cm^2^/m^2^ in men and < 39 cm^2^/m^2^ in women	Carey EJ (90)	Liver biopsy
Almeida (48)	NAFLD (+)	South America	cross-sectional	57	57	14 (24.6)	52.7	NA	2 (3.5)	LMM (BIA) + LMS (HGS) + LPP (4mGS)	BIA	SMM/ht^2^ < 8.87 kg/m^2^ in men and < 6.42 kg/m^2^ in women	EWGSOP (2010)	Abdominal US
Chung (20)	NAFLD (+)	Asia	cross-sectional	17,540	6,298	4,054 (64.4)	48.8	25.4	694 (11.0)	LMM (BIA)	BIA	ASM/wt (%) < 29.0 in men and < 22.9 in women	Kim YS (92)	Abdominal US
Chung (20)	NAFLD (−)	Asia	cross-sectional	17,540	11,242	4,618 (41.1)	45.5	22.1	261 (2.3)	LMM (BIA)	BIA	ASM/wt (%) < 29.0 in men and < 22.9 in women	Kim YS (92)	Abdominal US
Kang_wt (34)	NAFLD (+)	Asia	cross-sectional	10,711	10,711	5,661 (52.9)	47.9	23.9	615 (5.7)	LMM (BIA)	BIA	ASM/wt (%) < 29.0 in men and < 22.9 in women	Kim YS (92) Lee YH (93)	Abdominal US
Kang_BMI (34)	NAFLD (+)	Asia	cross-sectional	10,711	10,711	5,661 (52.9)	47.9	23.9	495 (4.6)	LMM (BIA)	BIA	ASM/BMI < 0.789 in men or <0.512 in women	FNIH	Abdominal US
Zhang (24)	NAFLD (+)	Asia	cross-sectional	1,112	369	197 (53.4)	53.5	23.4	151 (40.9)	LMM (BIA)	BIA	ASM/wt (%) < 32.2 in men and < 25.5 in women	Lee YH (94)	Abdominal US
Zhang (24)	NAFLD (−)	Asia	cross-sectional	1,112	743	444 (59.8)	53.4	22.0	139 (18.7)	LMM (BIA)	BIA	ASM/wt (%) < 32.2 in men and < 25.5 in women	Lee YH (94)	Abdominal US
Lee_GSHC (26)	NAFLD (+)	Asia	cross-sectional	9,691	4,168	2,730 (65.5)	51.2	26.1	1,288 (30.9)	LMM (BIA)	BIA	below the sex-specific lowest quintile of SMI	other	Abdominal US
Lee_GSHC (26)	NAFLD (−)	Asia	cross-sectional	9,691	5,523	2077 (37.6)	47.4	22.5	652 (11.8)	LMM (BIA)	BIA	below the sex-specific lowest quintile of SMI	other	Abdominal US
Petta (23)	NAFLD (+)	Europe	cross-sectional	225	225	141 (62.7)	48.3	30.3	98 (43.6)	LMM (BIA)	BIA	ASM/wt (%) < 37.0 in men and < 28.0 in women	Janssen I (89)	Liver biopsy
Seo_M_wt (28)	NAFLD (+)	Asia	cross-sectional	2,160	685	2,160 (95)	53.1	26.6	312 (45.5)	LMM (BIA)	BIA	ASM/wt (%) < 29.0 in men and < 22.9 in women	Kim KM (96)	Abdominal US
Seo_M_wt (28)	NAFLD (−)	Asia	cross-sectional	2,160	1,475	2,160 (95)	56.6	23.9	364 (24.7)	LMM (BIA)	BIA	ASM/wt (%) < 29.0 in men and < 22.9 in women	Kim KM (96)	Abdominal US
Seo_W_wt (28)	NAFLD (+)	Asia	cross-sectional	2050	593	0 (0)	58.9	26.4	216 (36.4)	LMM (BIA)	BIA	ASM/wt (%) < 29.0 in men and < 22.9 in women	Kim KM (96)	Abdominal US
Seo_W_wt (28)	NAFLD (−)	Asia	cross-sectional	2050	1,457	0 (0)	59.4	23.5	348 (23.9)	LMM (BIA)	BIA	ASM/wt (%) < 29.0 in men and < 22.9 in women	Kim KM (96)	Abdominal US
Seo_M_BMI (28)	NAFLD (+)	Asia	cross-sectional	2,160	685	2,160 (95)	53.1	26.6	241 (35.2)	LMM (BIA)	BIA	ASM/BMI < 0.789 in men or <0.512 in women	FNIH	Abdominal US
Seo_M_BMI (28)	NAFLD (−)	Asia	cross-sectional	2,160	1,475	2,160 (95)	56.6	23.9	356 (24.1)	LMM (BIA)	BIA	ASM/BMI < 0.789 in men or <0.512 in women	FNIH	Abdominal US
Seo_W_BMI (28)	NAFLD (+)	Asia	cross-sectional	2050	593	0 (0)	58.9	26.4	148 (25.0)	LMM (BIA)	BIA	ASM/BMI < 0.789 in men or <0.512 in women	FNIH	Abdominal US
Seo_W_BMI (28)	NAFLD (−)	Asia	cross-sectional	2050	1,457	0 (0)	59.4	23.5	246 (16.9)	LMM (BIA)	BIA	ASM/BMI < 0.789 in men or <0.512 in women	FNIH	Abdominal US
Wang_M (29)	NAFLD (+)	Asia	cross-sectional	92	30	30 (100)	68.9	25.7	6 (20.0)	LMM (DXA) + LMS (HGS)/LPP (4 m GS)	DXA	ASM/ht^2^ < 7.0 kg/m^2^ in men and <5.4 kg/m^2^ in women	AWGS (2014)	Abdominal US
Wang_M (29)	NAFLD (−)	Asia	cross-sectional	92	62	62 (100)	72.9	23.6	6 (9.7)	LMM (DXA) + LMS (HGS)/LPP (4 m GS)	DXA	ASM/ht^2^ < 7.0 kg/m^2^ in men and <5.4 kg/m^2^ in women	AWGS (2014)	Abdominal US
Wang_W (29)	NAFLD (+)	Asia	cross-sectional	486	124	0 (0)	67.5	24.7	19 (15.3)	LMM (DXA) + LMS (HGS)/LPP (4 m GS)	DXA	ASM/ht^2^ < 7.0 kg/m^2^ in men and <5.4 kg/m^2^ in women	AWGS (2014)	Abdominal US
Wang_W (29)	NAFLD (−)	Asia	cross-sectional	486	362	0 (0)	62.9	22.5	29 (8.0)	LMM (DXA) + LMS (HGS)/LPP (4 m GS)	DXA	ASM/ht^2^ < 7.0 kg/m^2^ in men and <5.4 kg/m^2^ in women	AWGS (2014)	Abdominal US
Zhao (42)	NAFLD (+)	North America	cross-sectional	13,692	4,207	2,108 (50.1)	48.3	30.7	2,659 (63.2)	LMM (BIA)	BIA	ASM/wt (%) < 37.0 in men and < 28.0 in women	Janssen I (97)	Abdominal US
Zhao (42)	NAFLD (−)	North America	cross-sectional	13,692	9,485	4,365 (46.0)	41.1	25.8	3,145 (33.2)	LMM (BIA)	BIA	ASM/wt (%) < 37.0 in men and < 28.0 in women	Janssen I (97)	Abdominal US
Choe (32)	NAFLD (+)	Asia	cross-sectional	1828	716	NA	NA	NA	238 (33.2)	LMM (CT)	CT	SMI < 8.37 cm^2^/(kg/m2) in men and 7.47 cm2/(kg/m^2^) in women	Janssen I (89)	Abdominal US
Choe (32)	NAFLD (−)	Asia	cross-sectional	1828	1,112	NA	NA	NA	216 (19.4)	LMM (CT)	CT	SMI < 8.37 cm^2^/(kg/m2) in men and 7.47 cm2/(kg/m^2^) in women	Janssen I (89)	Abdominal US
Golabi (38)	NAFLD (+)	North America	cross-sectional	4,611	1,351	811 (60.0)	50.8	32.5	239 (17.7)	LMM (DXA)	DXA	ASM/BMI < 0.789 in men or <0.512 in women	FNIH	USA FLI value
Golabi (38)	NAFLD (−)	North America	cross-sectional	4,611	3,260	1,413 (43.4)	43.9	25.8	183 (5.6)	LMM (DXA)	DXA	ASM/BMI < 0.789 in men or <0.512 in women	FNIH	USA FLI value
Gan (36)	NAFLD (+)	Asia	cross-sectional	3,536	1,088	358 (32.9)	55.2	25.9	246 (22.6)	LMM (DXA) + LMS (HGS)	DXA	ASM/wt (%) < 28.64 in men and < 24.12 in women	Other	Abdominal US
Gan (36)	NAFLD (−)	Asia	cross-sectional	3,536	2,448	658 (26.9)	51.7	22.1	119 (4.9)	LMM (DXA) + LMS (HGS)	DXA	ASM/wt (%) < 28.64 in men and < 24.12 in women	Other	Abdominal US
Kang (22)	NAFLD (+)	Asia	cross-sectional	433	49	31 (63.3)	45.1	23.0	25 (51.0)	LMM (CT)	CT	SMI < 545 mm^2^/m^2^ in men and < 385 mm^2^/m^2^ in women	Fearon K (98) Jones KI (95)	CT
Kang (22)	NAFLD (−)	Asia	cross-sectional	433	394	253 (64.2)	38.6	20.8	130 (33.0)	LMM (CT)	CT	SMI < 545 mm^2^/m^2^ in men and < 385 mm^2^/m^2^ in women	Fearon K (98) Jones KI (95)	CT
Park_wt (14)	NAFLD (+)	Asia	cross-sectional	1,343	747	509 (68.1)	48.9	24.9	66 (8.8)	LMM (BIA)	BIA	ASM/wt (%) < 29.1 in men and < 23.0 in women	Kim YS (92)	Abdominal US
Park_wt (14)	NAFLD (−)	Asia	cross-sectional	1,343	596	238 (31.9)	44.1	21.6	8 (1.3)	LMM (BIA)	BIA	ASM/wt (%) < 29.1 in men and < 23.0 in women	Kim YS (92)	Abdominal US
Park_BMI (14)	NAFLD (+)	Asia	cross-sectional	1,343	747	509 (68.1)	48.9	24.9	43 (5.8)	LMM (BIA)	BIA	ASM/BMI < 0.789 in men or <0.521 in women	Lee YH (99)	Abdominal US
Park_BMI (14)	NAFLD (−)	Asia	cross-sectional	1,343	596	238 (31.9)	44.1	21.6	8 (1.3)	LMM (BIA)	BIA	ASM/BMI < 0.789 in men or <0.521 in women	Lee YH (99)	Abdominal US
Park_ht^2^ (14)	NAFLD (+)	Asia	cross-sectional	1,343	747	509 (68.1)	48.9	24.9	6 (0.8)	LMM (BIA)	BIA	ASM/ht^2^ < 6.58 kg/m^2^ in men and <4.59 kg/m^2^ in women	Kim YS (92)	Abdominal US
Park_ht^2^ (14)	NAFLD (−)	Asia	cross-sectional	1,343	596	238 (31.9)	44.1	21.6	12 (2.0)	LMM (BIA)	BIA	ASM/ht^2^ < 6.58 kg/m^2^ in men and <4.59 kg/m^2^ in women	Kim YS (92)	Abdominal US
Jiang_M (21)	NAFLD (+)	Asia	cross-sectional	452	178	178 (100)	61.8	25.9	36 (20.2)	LMM (DXA)	DXA	ASM/wt (%) < 29.0 in men and < 22.9 in women	Janssen I (89)	Abdominal US
Jiang_M (21)	NAFLD (−)	Asia	cross-sectional	452	274	274 (100)	65.3	23.9	27 (9.9)	LMM (DXA)	DXA	ASM/wt (%) < 29.0 in men and < 22.9 in women	Janssen I (89)	Abdominal US
Jiang_W (21)	NAFLD (+)	Asia	cross-sectional	340	123	0 (0)	64.9	25.8	15 (12.2)	LMM (DXA)	DXA	ASM/wt (%) < 29.0 in men and < 22.9 in women	Janssen I (89)	Abdominal US
Jiang W (21)	NAFLD (−)	Asia	cross-sectional	340	217	0 (0)	65.7	24.4	11 (5.1)	LMM (DXA)	DXA	ASM/wt (%) < 29.0 in men and < 22.9 in women	Janssen I (89)	Abdominal US
Alferink_M (33)	NAFLD (+)	Europe	cross-sectional	1980	726	726 (100)	NA	NA	36 (4.9)	LMM (DXA) + LMS (HGS)/LPP (5.79 m GS)	DXA	ASM/ht^2^ < 7.25 kg/m^2^ in men and <5.67 kg/m^2^ in women	EWGSOP (2010)	Abdominal US
Alferink_M (33)	NAFLD (−)	Europe	cross-sectional	1980	1,254	1,254 (95)	NA	NA	94 (7.5)	LMM (DXA) + LMS (HGS)/LPP (5.79 m GS)	DXA	ASM/ht^2^ < 7.25 kg/m^2^ in men and <5.67 kg/m^2^ in women	EWGSOP (2010)	Abdominal US
Alferink_W (33)	NAFLD (+)	Europe	cross-sectional	2,629	897	0 (0)	NA	NA	12 (1.3)	LMM (DXA) + LMS (HGS)/LPP (5.79 m GS)	DXA	ASM/ht^2^ < 7.25 kg/m^2^ in men and <5.67 kg/m^2^ in women	EWGSOP (2010)	Abdominal US
Alferink_W (33)	NAFLD (−)	Europe	cross-sectional	2,629	1732	0 (0)	NA	NA	66 (3.8)	LMM (DXA) + LMS (HGS)/LPP (5.79 m GS)	DXA	ASM/ht^2^ < 7.25 kg/m^2^ in men and <5.67 kg/m^2^ in women	EWGSOP (2010)	Abdominal US
Chung (13)	NAFLD (+)	Asia	cross-sectional	5,989	2,290	NA	NA	NA	219 (9.6)	LMM (BIA)	BIA	ASM/wt (%) < 29.0 in men and < 22.9 in women	Kim YS (92)	Abdominal US
Chung (13)	NAFLD (−)	Asia	cross-sectional	5,989	3,699	NA	NA	NA	96 (2.6)	LMM (BIA)	BIA	ASM/wt (%) < 29.0 in men and < 22.9 in women	Kim YS (92)	Abdominal US
Pan (27)	NAFLD (+)	Asia	cross-sectional	401	401	401 (100)	38.8	27.1	201 (50.1)	LMM (BIA)	BIA	ASM/wt (%) < 30.6 in men	Other	Liver biopsy
Harring (49)	NAFLD (+)	North America	cross-sectional	2,422	1,056	578 (54.8)	41.9	33.5	304 (28.8)	LMM (DXA)	DXA	ASM/BMI < 0.789 in men or <0.512 in women	FNIH	Transient elastography
Harring (49)	NAFLD (−)	North America	cross-sectional	2,422	1,366	632 (46.3)	35.4	25.8	153 (11.2)	LMM (DXA)	DXA	ASM/BMI < 0.789 in men or <0.512 in women	FNIH	Transient elastography
Zhu (25)	NAFLD (+)	Asia	cohort	3,974	1,305	432 (33.1)	62.6	25.7	260 (19.9)	LMM (DXA)	DXA	ASM/ht^2^ < 6.88 kg/m^2^ in men and <5.67 kg/m^2^ in women	AWGS (2014)	Abdominal US
Sheptulina (41)	NAFLD (+)	Asia	cross-sectional	189	108	40 (37.0)	49.5	31	17 (15.7)	LPP (SARC-F questionnaire)	NA	NA	EWGSOP2 (2019)	Abdominal US
Amer (43)	NAFLD (+)	Asia	cross-sectional	159	159	57 (35.8)	NA	NA	71 (44.7)	LMM (BIA) + LMS (HGS)	BIA	ASM/wt (%) < 35.7 in men and < 30.7 in women	Lewandowicz A (100), FNIH	Liver biopsy
Zhang (31)	NAFLD (+)	Asia	cross-sectional	3,405	3,405	2,287 (67.2)	39.0	27.9	625 (18.4)	LMM (DXA)	DXA	ASM/BMI < 0.789 in men or <0.512 in women	FNIH	Transient elastography
Cho_wt (40)	NAFLD (+)	Asia	cohort	852	456	211 (46.3)	55.1	25.8	123 (27.0)	LMM (BIA)	BIA	ASM/wt (%) < 29.0 in men and < 22.9 in women	Kim KM (96)	Abdominal US
Cho_wt (40)	NAFLD (−)	Asia	cohort	852	396	172 (43.4)	55.8	23.3	66 (16.7)	LMM (BIA)	BIA	ASM/wt (%) < 29.0 in men and < 22.9 in women	Kim KM (96)	Abdominal US
Cho_ht^2^ (40)	NAFLD (+)	Asia	cohort	852	456	211 (46.3)	55.1	25.8	89 (19.5)	LMM (BIA)	BIA	ASM/ht^2^ < 7.0 kg/m^2^ in men and < 5.7 kg/m^2^ in women	AWGS (2014)	Abdominal US
Cho_ht^2^ (40)	NAFLD (−)	Asia	cohort	852	396	172 (43.4)	55.8	23.3	116 (29.3)	LMM (BIA)	BIA	ASM/ht^2^ < 7.0 kg/m^2^ in men and < 5.7 kg/m^2^ in women	AWGS (2014)	Abdominal US
Seo_wt (39)	NAFLD (+)	Asia	cross-sectional	6,414	3,198	2,873 (89.8)	54.2	26.2	517 (16.2)	LMM (BIA)	BIA	ASM/wt (%) < 29.1 in men and < 23.0 in women	Kim YS (92)	Transient elastography
Seo_wt (39)	NAFLD (−)	Asia	cross-sectional	6,414	3,216	2,604 (81.0)	53.7	23.1	94 (2.9)	LMM (BIA)	BIA	ASM/wt (%) < 29.1 in men and < 23.0 in women	Kim YS (92)	Transient elastography
Seo_BMI (39)	NAFLD (+)	Asia	cross-sectional	6,414	3,198	2,873 (89.8)	54.2	26.2	277 (8.7)	LMM (BIA)	BIA	ASM/BMI < 0.789 in men or <0.512 in women	FNIH	Transient elastography
Seo_BMI (39)	NAFLD (−)	Asia	cross-sectional	6,414	3,216	2,604 (81.0)	53.7	23.1	117 (3.6)	LMM (BIA)	BIA	ASM/BMI < 0.789 in men or <0.512 in women	FNIH	Transient elastography
Lee_ht^2^ (26)	NAFLD (+)	Asia	cross-sectional	320	183	68 (37.2)	65.7	26.9	57 (31.1)	LMM (DXA)	DXA	ASM/ht^2^ < 7.0 kg/m^2^ in men and < 5.4 kg/m^2^ in women	AWGS (2014)	Abdominal US
Lee_ht^2^ (26)	NAFLD (−)	Asia	cross-sectional	320	137	39 (28.5)	67.2	23.5	87 (63.5)	LMM (DXA)	DXA	ASM/ht^2^ < 7.0 kg/m^2^ in men and < 5.4 kg/m^2^ in women	AWGS (2014)	Abdominal US
Lee_BMI (26)	NAFLD (+)	Asia	cross-sectional	320	137	39 (28.5)	67.2	23.5	107 (58.5)	LMM (DXA)	DXA	ASM/BMI < 0.789 in men or <0.512 in women	FNIH	Abdominal US
Lee_BMI (26)	NAFLD (−)	Asia	cross-sectional	320	137	39 (28.5)	67.2	23.5	72 (52.6)	LMM (DXA)	DXA	ASM/BMI < 0.789 in men or <0.512 in women	FNIH	Abdominal US
Lee_wt (26)	NAFLD (+)	Asia	cross-sectional	320	137	39 (28.5)	67.2	23.5	148 (80.9)	LMM (DXA)	DXA	ASM/wt (%) < 29.0 in men and < 22.9 in women	Kim YS (92)	Abdominal US
Lee_wt (26)	NAFLD (−)	Asia	cross-sectional	320	137	39 (28.5)	67.2	23.5	87 (63.5)	LMM (DXA)	DXA	ASM/wt (%) < 29.0 in men and < 22.9 in women	Kim YS (92)	Abdominal US

NAFLD (+), patients with nonalcoholic fatty liver; NAFLD (−), patients without nonalcoholic fatty liver; BMI, body mass index; BIA, bioelectrical impedance analysis; DXA, dual-energy X-ray absorptiometry; CT, computed tomography; MRI, magnetic resonance imaging; abdominal US, abdominal ultrasound; LMM, low muscle mass; LMS, low muscle strength; LPP, low physical performance; ASM, appendicular skeletal muscle mass; HGS, hand grip strength; GS, gait speed; SMI, skeletal muscle mass index; SARC-F, a simple five-item questionnaire; wt: weight; ht^2^: height square; aORs: adjusted odds ratios; HSI, hepatic steatosis index; USA FLI, fatty liver index for the multi-ethnic US population; AWGS, Asian Working Group for Sarcopenia; EWGSOP, European Working Group on Sarcopenia in Older People; EWGSOP2, European Working Group on Sarcopenia in Older People 2; FNIH: Foundation for the National Institutes of Health Sarcopenia Project; NA, not applicable. Other: sarcopenia diagnostic criteria other than EWGSOP (2010), EWGSOP2 (2019), AWGS (2014), AWGS (2019), and FNIH.

a Mean as reported.

**Table 2:** Characteristics of the included studies for adjusted ORs between sarcopenia and NAFLD and liver fibrosis.

First author and year	Study region	Study design	Sample size	Number of NAFLD	Male, n (%)	Age^a^ (years)	BMI^a^ (kg/m^2^)	Criteria (assessment method to detect sarcopenia)	Cut-off value for muscle mass	Sarcopenia diagnostic criteria	NAFLD diagnosis	aORs for NAFLD	Fibrosis diagnosis	aORs for fibrosis
Hong (51)	Asia	cohort	452	NA	NA	NA	NA	LMM (DXA)	ASM/wt (%) < 39.8 in men and < 34.1 in women	Janssen I (89)	CT	5.16 (1.63, 16.3)	NA	NA
Hashimoto_M (56)	Asia	cross-sectional	79	58	58 (100)	63.2	25.7	LMM (BIA)	NA	other	Transient elastography	0.80 (0.64, 0.97)	NA	NA
Hashimoto_W (56)	Asia	cross-sectional	66	39	0 (0)	62.4	26.3	LMM (BIA)	NA	other	Transient elastography	0.97 (0.81,1.14)	NA	NA
Kim_M (52)	Asia	cross-sectional	1,184	208	208 (100)	44.0	28	LMM (DXA)	NA	Janssen I (89)	FLI value	1.35 (1.17, 1.54)	NA	NA
Kim_W (52)	Asia	cross-sectional	2,555	181	181 (0)	55.6	30.1	LMM (DXA)	NA	Janssen I (89)	FLI value	1.36 (1.18, 1.55)	NA	NA
Hong_wt (35)	Asia	cross-sectional	850	124	83 (66.9)	63	27	LMM (DXA)	ASM/wt (%) < 29.0 in men and < 22.9 in women	Koo BK (101) Lee YH (93)	HSI value	2.25 (1.30, 3.92)	NA	NA
Hong_BMI (35)	Asia	cross-sectional	850	124	83 (66.9)	63	27	LMM (DXA)	ASM/BMI < 0.789 in men or <0.512 in women	FNIH	HSI value	1.95 (1.11, 3.46)	NA	NA
Hong_ht^2^ (35)	Asia	cross-sectional	850	124	83 (66.9)	63	27	LMM (DXA)	ASM/ht^2^ < 7.0 kg/m^2^ in men and < 5.4 kg/m^2^	AWGS (2014)	HSI value	1.06 (0.67, 1.68)	NA	NA
Song (55)	Asia	cross-sectional	2,191	1,180	844 (71.5)	53.3	26.7	LMM (BIA)	ASM/wt (%) < 30.0 in men and < 26.8 in women	Kim TN (102)	Abdominal US	1.92 (1.30, 2.84)	Transient elastography	SF: 3.80 (0.86, 16.75)
Kim_M (54)	Asia	cross-sectional	14,400	3,138	3,138 (100)	53.0	25.8	LMM (CT)	SMI/BMI below one SD (20–44 years)	Kim EH (103)	Abdominal US	1.27 (1.10, 1.47)	FIB-4	NA
Kim_W (54)	Asia	cross-sectional	14,400	1,610	1,610 (0)	56.1	25.0	LMM (CT)	SMI/BMI below one SD (20–44 years)	Kim EH (103)	Abdominal US	1.42 (1.26, 1.60)	FIB-4	NA
Zhang (88)	Asia	cross-sectional	1,112	369	197 (53.4)	53.5	23.4	LMM (BIA)	ASM/wt (%) < 32.2 in men and < 25.5 in women	Lee YH (94)	Abdominal US	2.07 (1.23, 3.46)	NA	NA
Lee_GSHC (26)	Asia	cross-sectional	9,691	4,168	2,730 (65.5)	51.2	26.1	LMM (BIA)	the sex-specific lowest quintile of SMI	other	Abdominal US	2.76 (2.34, 3.27)	NA	NA
Lee_KNHANES (26)	Asia	cross-sectional	8,320	1977	980 (49.6)	51.0	25.6	LMM (DXA)	ASM/BMI < 0.789 in men or <0.512 in women	FNIH	NAFLD liver fat score	1.67 (1.08, 2.56)	NA	NA
Lee_koGES (26)	Asia	cohort	4,587	1,008	476 (47.2)	52.3	26.0	LMM (BIA)	the sex-specific lowest quintile of SMI	other	NAFLD liver fat score	1.57 (1.35, 1.83)	NA	NA
Seo_M_wt (62)	Asia	cross-sectional	2,160	685	685 (100)	53.1	26.6	LMM (BIA)	ASM/wt (%) < 29.0 in men and < 22.9 in women	Kim KM (96)	Abdominal US	1.58 (1.15, 2.17)	NA	NA
Seo_W_wt (62)	Asia	cross-sectional	2050	593	0 (0)	58.9	26.4	LMM (BIA)	ASM/wt (%) < 29.0 in men and < 22.9 in women	Kim KM (96)	Abdominal US	0.97 (0.71, 1.38)	NA	NA
Seo_M_BMI (62)	Asia	cross-sectional	2,160	685	685 (100)	53.1	26.6	LMM (BIA)	ASM/BMI < 0.789 in men or < 0.512 in women	FNIH	Abdominal US	1.41 (1.02, 1.94)	NA	NA
Seo_W_BMI (62)	Asia	cross-sectional	2050	593	0 (0)	58.9	26.4	LMM (BIA)	ASM/BMI < 0.789 in men or < 0.512 in women	FNIH	Abdominal US	1.06 (0.75, 1.52)	NA	NA
Wang_M (29)	Asia	cross-sectional	92	30	30 (100)	68.9	25.7	LMM (DXA) + LMS (HGS)+/LPP (4 m GS)	ASM/ht^2^ < 7.0 kg/m^2^ in men and < 5.4 kg/m^2^	AWGS (2014)	Abdominal US	1.33 (1.03, 1.73)	NA	NA
Wang_W (29)	Asia	cross-sectional	486	124	0 (0)	67.5	24.7	LMM (DXA) + LMS (HGS)+/LPP (4 m GS)	ASM/ht^2^ < 7.0 kg/m^2^ in men and < 5.4 kg/m^2^	AWGS (2014)	Abdominal US	1.37 (1.11, 1.69)	NA	NA
Wijarnpreecha (30)	North America	cross-sectional	11,325	4,188	2,111 (50.4)	45.4	29.0	LMM (BIA)	ASM/wt (%) < 37.0 in men and < 28.0 in women	Janssen I (89)	Abdominal US	1.24 (1.04, 1.48)	NAFLD fibrosis score	AF: 1.79 (1.18, 2.72)
Choe (32)	Asia	cross-sectional	1828	716	NA	NA	NA	LMM (CT)	SMI < 8.37 cm^2^/(kg/m^2^) in men and < 7.47 cm^2^/(kg/m^2^) in women	Janssen I (89)	Abdominal US	1.51 (1.15, 1.99)	NA	NA
Gan (36)	Asia	cross-sectional	3,536	1,088	358 (32.9)	55.2	25.9	LMM (DXA) + LMS (HGS)	ASM/wt (%) < 28.64 in men and < 24.12 in women	other	Abdominal US	3.91 (2.90, 5.28)	NA	NA
Kang (22)	Asia	cross-sectional	433	49	31 (63.3)	45.1	23.0	LMM (CT)	SMI < 545 mm^2^/m^2^ in men and < 385 mm^2^/m^2^ in women	Fearon K (98) Jones KI (95)	CT	2.26 (1.15, 4,42)	NA	NA
Park (14)	Asia	cross-sectional	1,343	747	509 (68.1)	48.9	24.9	LMM (BIA)	ASM/wt (%) < 29.1 in men and < 23.0 in women	Kim YS (92)	Abdominal US	0.95 (0.83, 1.08)	NA	NA
Jiang_M (21)	Asia	cross-sectional	452	178	1	61.8	25.9	LMM (DXA)	ASM/wt (%) < 29.0 in men and < 22.9 in women	Janssen I (89)	Abdominal US	2.01 (1.01, 3.98)	NA	NA
Jiang_W (21)	Asia	cross-sectional	340	123	0	64.9	25.8	LMM (DXA)	ASM/wt (%) < 29.0 in men and < 22.9 in women	Janssen I (89)	Abdominal US	1.70 (0.64, 4.51)	NA	NA
Alferink_M (33)	European	cross-sectional	4,609	726	1	NA	NA	LMM (DXA) + LMS (HGS) or LPP (5.79 m GS)	ASM/ht^2^ < 7.25 kg/m^2^ in men and < 5.67 kg/m^2^	EWGSOP (2010)	Abdominal US	1.96 (1.03, 3.72)	Transient elastography	NA
Alferink_W (33)	European	cross-sectional	4,609	897	0	NA	NA	LMM (DXA) + LMS (HGS) or LPP (5.79 m GS)	ASM/ht^2^ < 7.25 kg/m^2^ in men and < 5.67 kg/m^2^	EWGSOP (2010)	Abdominal US	0.84 (0.37, 1.90)	Transient elastography	NA
Chung (13)	Asia	cross-sectional	5,989	2,290	NA	NA	NA	LMM (BIA)	ASM/wt (%) < 29.0 in men and < 22.9 in women	Kim YS (92)	Abdominal US	1.37 (1.02, 1.84)	NA	NA
Chung (20)	Asia	cross-sectional	17,540	6,298	4,054 (64.4)	48.8	25.4	LMM (BIA)	ASM/wt (%) < 29.0 in men and < 22.9 in women	Kim YS (92)	Abdominal US	3.53 (3.01, 4.14)	NAFLD fibrosis score	AF: 1.32 (1.07, 1.61)
Pan (27)	Asia	cross-sectional	401	401	401 (100)	38.8	27.1	LMM (BIA)	ASM/wt (%) ≤ 30.6 in men	other	Liver biopsy	1.65 (1.30, 2.10)	Liver biopsy	AF: 1.24 (0.72, 2.14)
Guo (53)	Asia	cross-sectional	3,602	1830	1,467 (80.2)	47.4	27.1	LMM (BIA)	NA	other	Abdominal US	3.80 (2.20, 6.70)	Transient elastography	SF: 3.70 (2.60, 5.30)
Kang_wt (34)	Asia	cross-sectional	10,711	10,711	5,661 (52.9)	47.9	23.9	LMM (BIA)	ASM/wt (%) < 29.0 in men and < 22.9 in women	Lee YH (93) Kim YS (92)	Abdominal US	NA	NAFLD fibrosis score	AF: 2.68 (1.28, 5.59)
Kang_BMI (34)	Asia	cross-sectional	10,711	10,711	5,661 (52.9)	47.9	23.9	LMM (BIA)	ASM/BMI < 0.789 in men or < 0.512 in women	FNIH	Abdominal US	NA	NAFLD fibrosis score	AF: 3.12 (1.51, 6.46)
Petta (23)	Europe	cross-sectional	225	225	141 (62.7)	48.3	30.3	LMM (BIA)	ASM/wt (%) < 37.0 in men and < 28.0 in women	Janssen I (89)	Liver biopsy	NA	Liver biopsy	AF: 2.36 (1.16, 4.77)
Zhu (25)	Asia	cohort	3,974	1,305	432 (33.1)	62.6	25.7	LMM (DXA)	ASM/ht^2^ < 6.88 kg/m^2^ in men and < 5.67 kg/m^2^	AWGS (2014)	Abdominal US	NA	FIB-4	AF: 2.07 (1.24, 3.44)
Zhang_NHANES (31)	North America	cross-sectional	821	821	415 (50.6)	43.0	31.7	LMM (DXA)	ASM/BMI < 0.789 in men or < 0.512 in women	FNIH	Transient elastography	NA	Transient elastography	AF: 2.83 (1.55, 5.16)
Zhang (31)	Asia	cross-sectional	3,405	3,405	2,287 (67.2)	39.0	27.9	LMM (DXA)	ASM/BMI < 0.789 in men or < 0.512 in women	FNIH	Transient elastography	NA	Transient elastography	AF: 1.27 (1.01, 1.60)

BMI, body mass index; BIA, bioelectrical impedance analysis; DXA, dual-energy X-ray absorptiometry; CT, computed tomography; abdominal US, abdominal ultrasound; LMM, low muscle mass; LMS, low muscle strength; LPP, low physical performance; ASM, appendicular skeletal muscle mass; HGS, hand grip strength; GS, gait speed; SMI, skeletal muscle mass index; wt: weight; ht^2^: height square; aORs: adjusted odds ratios; FLI, fatty liver index; HSI, hepatic steatosis index; FIB-4, fibrosis 4 score; SF, significant fibrosis; AF, advanced fibrosis; AWGS, Asian Working Group for Sarcopenia; EWGSOP, European Working Group on Sarcopenia in Older People; FNIH: Foundation for the National Institutes of Health Sarcopenia Project; NA, not applicable. Other: sarcopenia diagnostic criteria other than EWGSOP (2010), EWGSOP2 (2019), AWGS (2014), AWGS (2019), and FNIH.

a Mean as reported.

### 2.5 Assessment of methodological quality

Two independent reviewers assessed study quality using the National Institutes of Health (NIH) Quality Assessment Tool for Observational Cohort and Cross-Sectional Studies (18), which evaluates 14 methodological criteria. For prevalence studies, we applied a validated 10-item checklist to examine the measurement validity, participant selection, and analytical methods (19). Studies were categorized as having low (score ≥8), moderate (6, 7), or high-risk (≤5) bias. Discrepancies were resolved through a discussion with a third reviewer.

### 2.6 Statistical analyses

We employed a random-effects model to estimate the pooled prevalence and total relative risk with 95% CI, optimizing the control for Type I error. All analyses were conducted using STATA 17.0 (StataCorp, College Station, TX, United States), with forest plots visualizing study-specific and pooled estimates for sarcopenia prevalence and NAFLD/liver fibrosis risk stratification. Multivariate aORs were preferentially extracted when available to maximize confounding control. Heterogeneity was assessed using I^2^ statistics (>50%) and Cochran’s Q-test (*p* < 0.10). Prespecified subgroup analyses (by region, study design, sarcopenia criteria, muscle mass assessment, and NAFLD/fibrosis evaluation method) explored potential sources of heterogeneity. Sensitivity analyses examined robustness by sequentially excluding individual studies, while publication bias was evaluated via funnel plots and Egger’s/Begg’s tests.

## 3 Results

### 3.1 Search process

**Figure 1:**
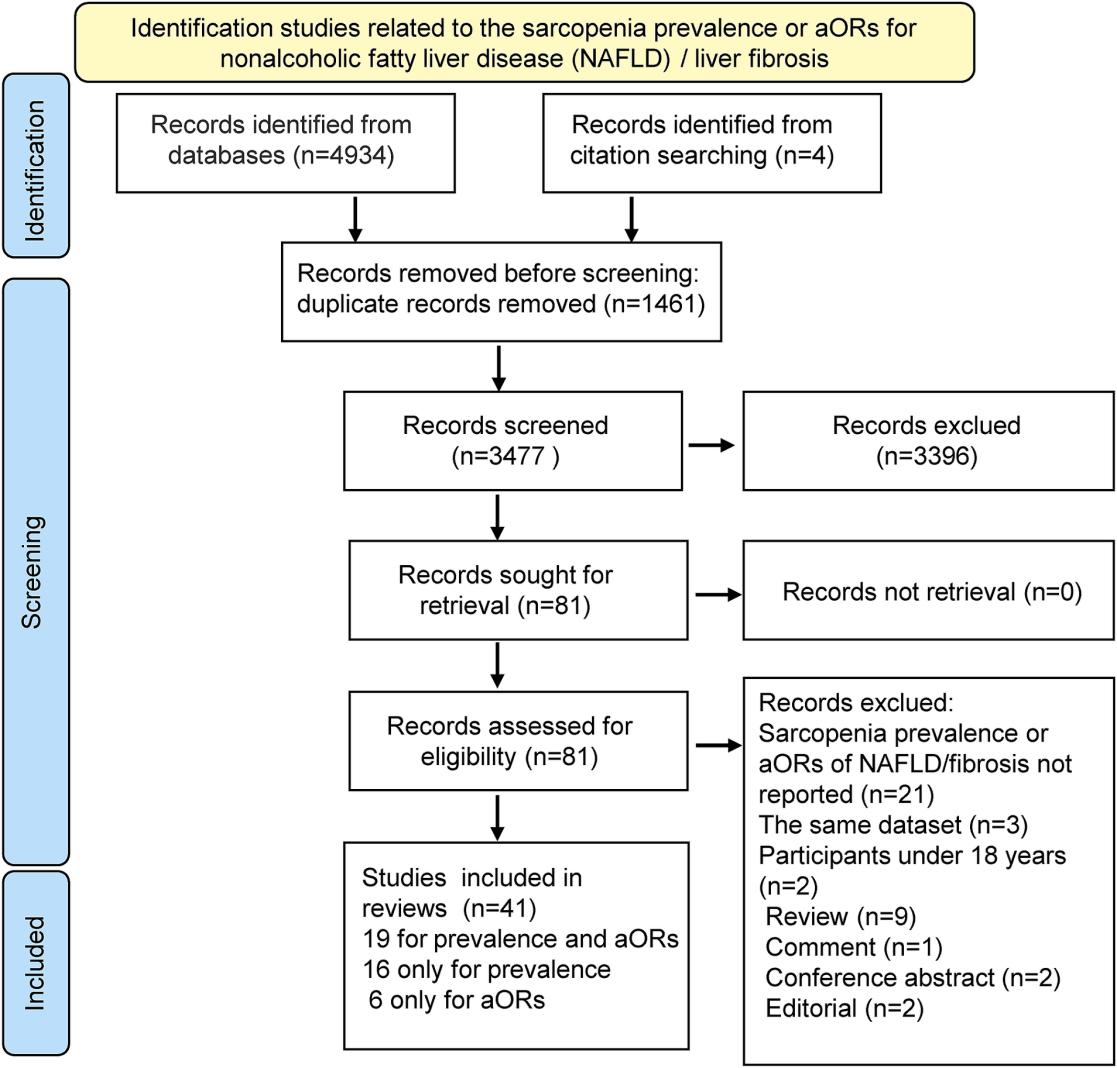
The flow chart of the literature selection. aORs, adjusted odds ratios.

A total of 4,938 articles related to sarcopenia and NAFLD were identified in the databases after a systematic search. Following deduplication, the titles and abstracts of 3,477 articles were screened and read, and 3,396 irrelevant studies were removed. Of the 81 articles selected for full-text evaluation, 40 were excluded based on the predefined criteria (Figure 1; Supplementary Table S3).

Finally, 41 articles involving 185,575 participants were included in our review, among which 19 (13, 14, 20–36) articles investigated prevalence and aORs, 16 (12, 15, 37–50) reported only prevalence, and 6 (51–56) reported only aORs.

### 3.2 Study characteristics

Characteristics are presented for studies included in each meta-analysis: 33 studies provided prevalence data, 21 studies reported aORs for NAFLD risk, and 9 studies reported aORs for liver fibrosis. Our analysis included 33 studies (2016–2025) with 135,143 participants (54,975 NAFLD cases; 29,969 males), showing sarcopenia prevalence ranging from 0.8 to 80.9% across ages 38.8 to 68.9 years. Most studies were cross-sectional (41 studies) and used various assessment methods, such as bioelectrical impedance analysis (25 studies), dual-energy X-ray absorptiometry (16), and computed tomography (4). Skeletal muscle index (SMI) normalization methods included weight (17 studies), height^2^ (12), and BMI (12). NAFLD diagnosis primarily used ultrasound (32 studies), with some employing elastography (4) or biopsy (4). Geographically, the studies were predominantly Asian (*n* = 35), European (*n* = 6), North American (*n* = 4), and South American (*n* = 1).

The characteristics of the studies that reported aORs are summarized in Table 2. Among the 21 studies assessing NAFLD risk in sarcopenia patients (2014–2023, *N* = 101,673; 35,023 NAFLD cases, 20,115 males), five applied comprehensive sarcopenia definitions, while 29 used non-comprehensive criteria. NAFLD diagnosis predominantly relies on abdominal US (22 studies), with fewer studies employing transient elastography (2), computed tomography (CT; 2), liver biopsy (1), or alternative methods (7; e.g., fatty liver index). For liver fibrosis (9 studies, 2016–2025, *N* = 54,195; 30,364 NAFLD cases, 17,813 males), diagnostic tools included transient elastography (4 studies), NAFLD fibrosis score (4), liver biopsy (2), and FIB-4 (1). All fibrosis studies adopted non-comprehensive definitions of sarcopenia.

### 3.3 Risk of bias in the included studies

Quality assessment using the Quality Assessment NIH tool classified 33 studies as high-quality and eight as moderate-quality (Supplementary Table S4). In addition, the prevalence-specific evaluation identified 23 studies with a low risk and 10 with a moderate risk of bias (Supplementary Table S5).

### 3.4 Synthesis with meta-analysis

#### 3.4.1 The overall prevalence of sarcopenia in patients with NAFLD

**Figure 2:**
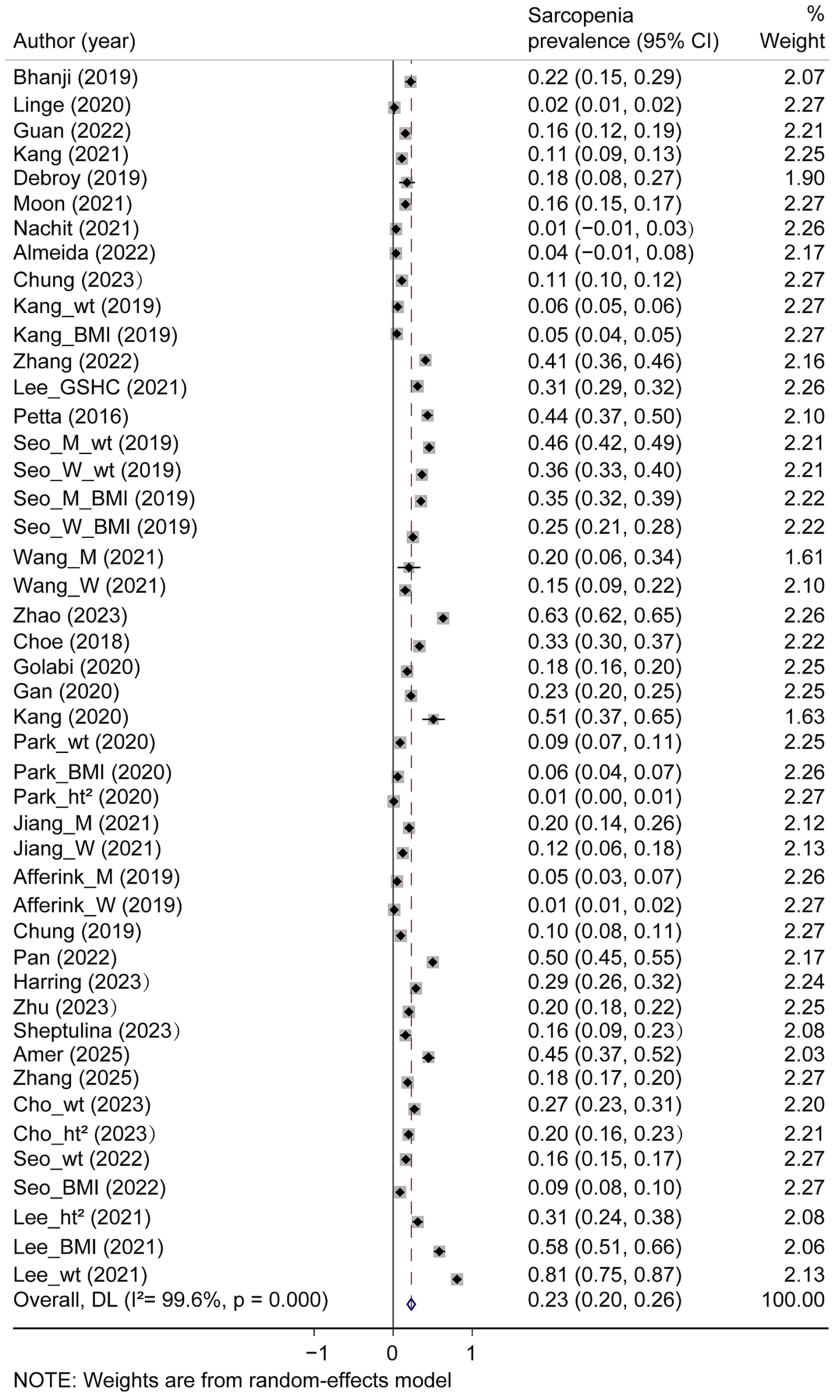
The pooled estimated prevalence rates of sarcopenia in nonalcoholic fatty liver disease patients.

Among the 33 studies that evaluated patients with NAFLD, the prevalence of sarcopenia demonstrated substantial heterogeneity, ranging from 0.8 to 80.9%. The pooled prevalence estimate was significantly higher in patients with NAFLD (23, 95% CI: 20 to 26%; Figure 2) than in non-NAFLD controls (15, 95% CI: 13 to 17%; Supplementary Figure S1).

#### 3.4.2 Subgroup analyses

Our analysis was stratified according to the different assessment tools for skeletal muscle mass. The pooled prevalence of sarcopenia in patients with NAFLD was 23% (95% CI: 18 to 27%) using BIA, 24% (95% CI: 18 to 29%) using DXA, and 26% (95% CI: 5 to 48%) using CT. The results revealed no notable differences in the prevalence of sarcopenia among the different muscle assessment techniques. However, normalization approaches showed marked variation: weight-adjusted SMI (SMI_wt) demonstrated a substantially higher prevalence (32, 95% CI: 23 to 40%) than height-squared normalization (SMI_ht^2^; 11, 95% CI: 8 to 14%), BMI-adjusted (SMI_BMI: 21, 95% CI: 16 to 27%), and CT-based (SMI_CT; 26, 95% CI: 5 to 48%). Diagnostic criteria significantly affected estimates, with comprehensive criteria yielding a lower prevalence (6, 95% CI: 4 to 9%) than non-comprehensive approaches (26, 95% CI: 22 to 29%). NAFLD diagnostic methods showed moderate variability in the prevalence of sarcopenia: abdominal US (23, 95% CI: 19 to 27%), transient elastography (24, 95% CI: 15 to 32%), liver biopsy (28, 95% CI: 12 to 44%), and other techniques (19, 95% CI: 11 to 28%). Geographical analysis revealed significant regional disparities, with Asia demonstrating a substantially higher prevalence of sarcopenia (24, 95% CI: 21 to 27%) than Europe (9, 95% CI: 5 to 12%) and South America (4, 95% CI: 0 to 8%). While North America showed the highest point estimate (33, 95% CI: 6 to 60%), the wide confidence intervals precluded definitive statistical comparisons with other regions (Supplementary Table S6).

#### 3.4.3 Publication bias and sensitivity analysis

Visual inspection of the funnel plot revealed asymmetry (Supplementary Figure S2), which was supported by a significant Egger test (*p* < 0.001), whereas the Begg test showed no statistical significance (*p* = 0.125). To assess the potential publication bias, we performed a trim-and-fill analysis, which identified no missing studies on either side of the plot. The pooled effect estimate remained stable, indicating minimal publication bias for overall prevalence (Supplementary Figure S3). Sensitivity analysis further confirmed the robustness of our findings, as the estimated prevalence of sarcopenia in patients with NAFLD was not substantially altered by sequential exclusion of any individual study (Supplementary Figure S4).

#### 3.4.4 Overall results of odds ratios for NAFLD and liver fibrosis in sarcopenia patients

**Figure 3:**
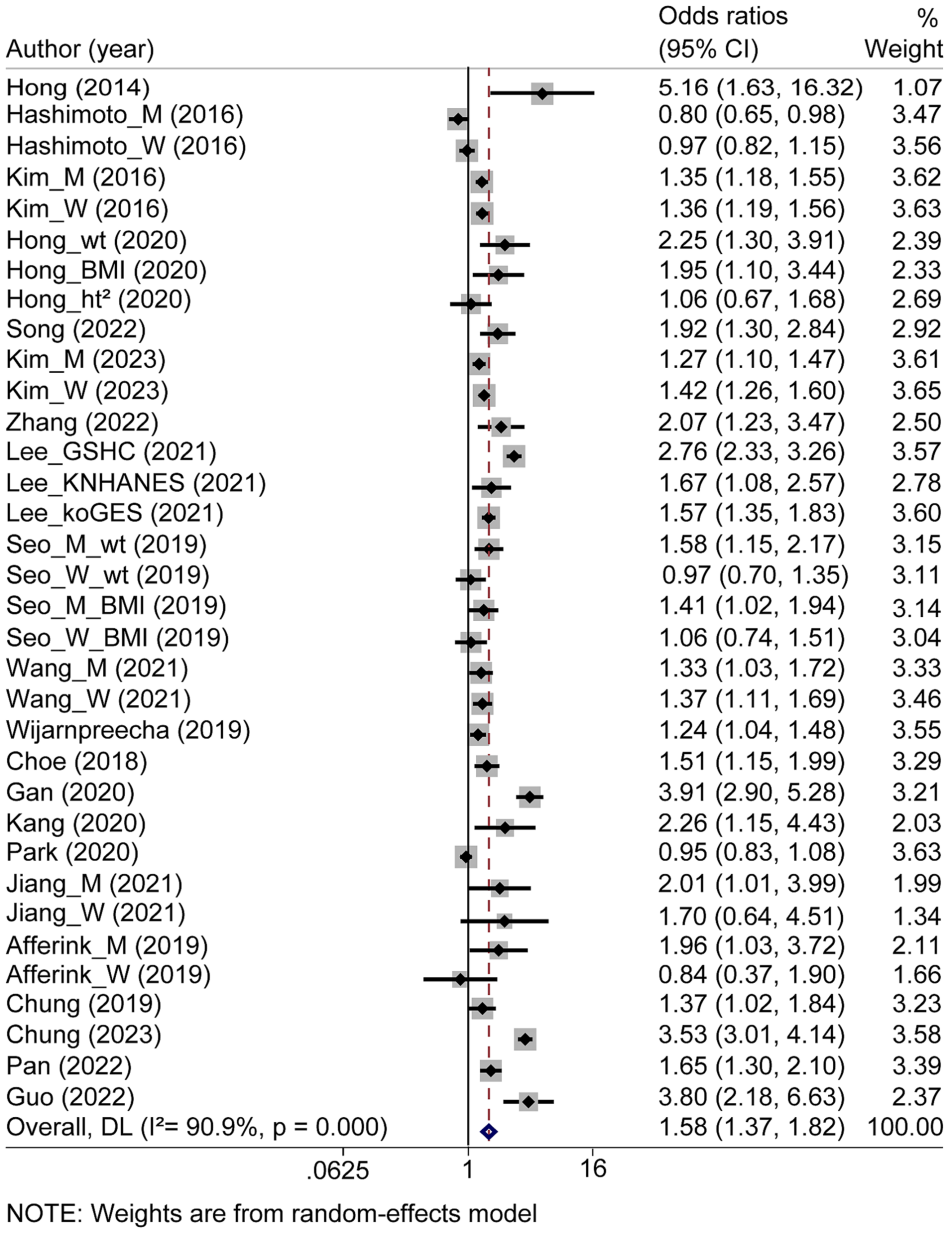
Forest plot of the overall adjusted odds ratios for nonalcoholic fatty liver disease in sarcopenia patients. CI, confidence interval.

**Figure 4:**
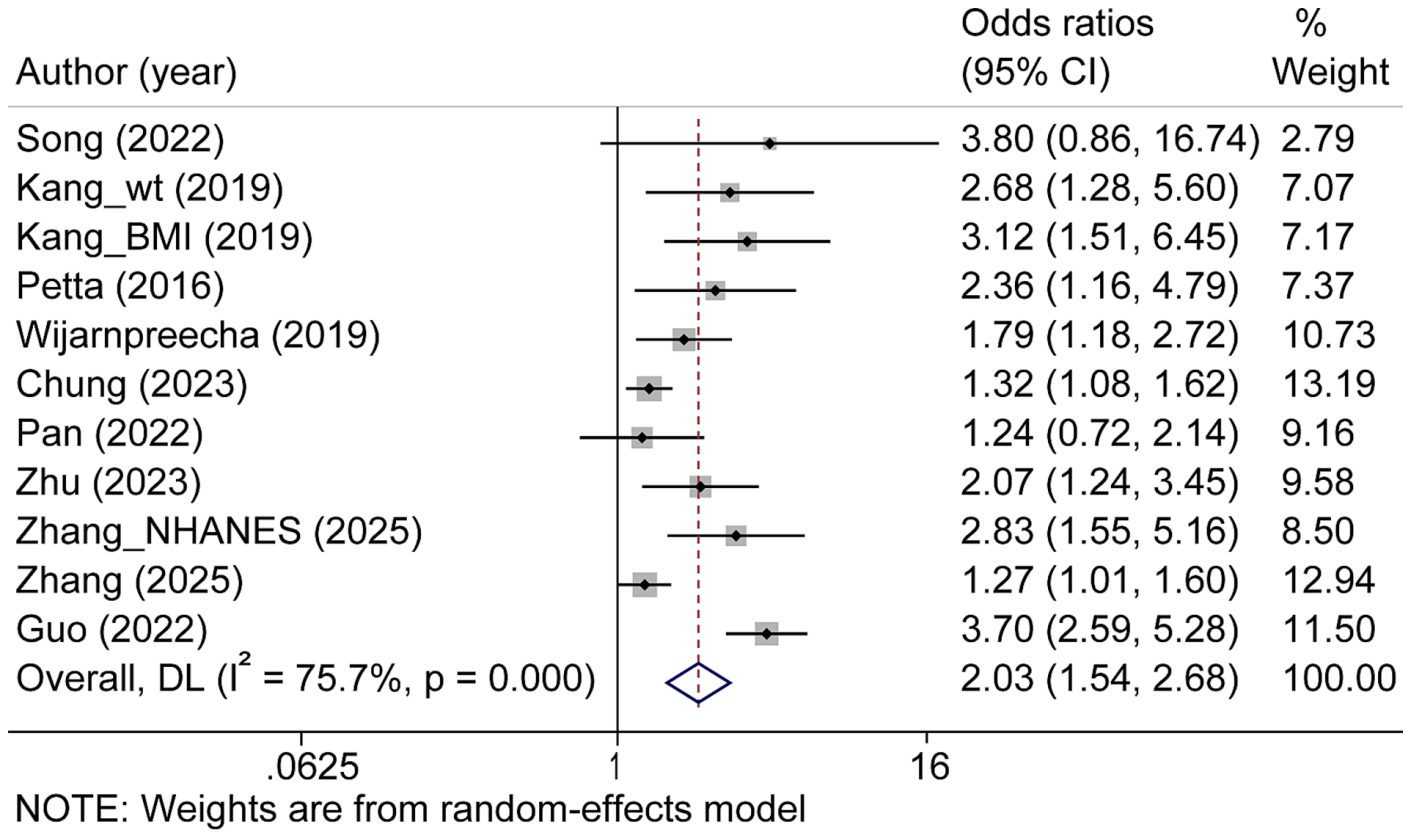
Forest plot of the overall adjusted odds ratios for liver fibrosis in sarcopenia patients. CI, confidence interval.

Among the 21 studies, 101,673 and 35,023 patients among the 21 studies. The overall aORs for NAFLD in patients with sarcopenia was 1.58 (95% CI: 1.37 to 1.82; Figure 3) across the studies (*p* < 0.001; *I^2^* = 90.9%). Among 49,611 subjects from eight studies, sarcopenia was associated with a higher risk of liver fibrosis in patients with NAFLD, with an overall aORs of 2.03 (95% CI: 1.54 to 2.68; Figure 4). The aORs was 2.44 (95% CI: 1.02 to 5.83) for significant liver fibrosis and 1.84 (95% CI: 1.44 to 2.34) for advanced liver fibrosis (Supplementary Table S7). Moderate heterogeneity in both aORs (*p* < 0.001; *I^2^* = 75.7%) was observed among these studies.

#### 3.4.5 Subgroup analyses

Subgroup analyses using different assessment methods revealed important patterns in the association between sarcopenia and NAFLD/liver fibrosis risk. For NAFLD risk, muscle mass assessment techniques showed consistent associations with BIA (aORs = 1.53, 95% CI: 1.19 to 1.96), DXA (aORs = 1.67, 95% CI: 1.37 to 2.03), and CT (aORs = 1.39, 95% CI: 1.25 to 1.55), with no significant inter-method differences. Normalization methods yielded similar results: SMI_wt (aORs = 1.66, 95% CI: 1.33 to 2.09), SMI_ht^2^ (1.67, 95% CI: 1.23 to 2.28), SMI_BMI (1.33, 95% CI: 1.14 to 1.54), and SMI_CT (1.39, 95% CI: 1.25 to 1.55). Stratification by diagnostic criteria revealed differential associations between sarcopenia and NAFLD risk, with comprehensively defined cases demonstrating an aORs of 1.70 (95% CI: 1.04 to 2.79) compared to 1.55 (95% CI: 1.34 to 1.80) for non-comprehensive classifications. Intriguingly, diagnostic modality-specific analyses yielded heterogeneous effect estimates: abdominal US produced an aORs of 1.64 (95% CI: 1.35 to 2.00), transient elastography showed a non-significant association (aORs = 0.89, 95% CI: 0.74 to 1.07), while CT-based assessment revealed the strongest effect magnitude (aORs = 2.98, 95% CI: 1.39 to 6.38), followed by liver biopsy (aORs = 1.65, 95% CI: 1.30 to 2.10). Geographical stratification identified Asia as exhibiting the most robust association (aORs = 1.60, 95% CI: 1.38 to 1.86), with more modest effects in the European (aOR = 1.33, 95% CI: 0.58 to 3.05) and North American cohorts (aOR = 1.24, 95% CI: 1.04 to 1.48).

For liver fibrosis, muscle mass quantification methodologies revealed distinct effect estimates for the sarcopenia-liver fibrosis association, with BIA demonstrating an aORs of 2.15 (95% CI: 1.48 to 3.13) compared to DXA 1.84 (95% CI: 1.12 to 3.03). Normalization approaches yielded remarkably consistent risk estimates across modalities: SMI_wt produced an aORs of 2.06 (95% CI: 1.38 to 3.07), SMI_ht^2^ 2.07 (95% CI: 1.24 to 3.45), and SMI_BMI 2.10 (95% CI: 1.08 to 4.08). Fibrosis assessment techniques exhibited greater variability in effect sizes, ranging from transient elastography’s robust association (aORs = 2.49, 95% CI: 1.22 to 5.05) to liver biopsy’s more-modest estimate (aORs = 1.64, 95% CI: 0.88 to 3.07). The NAFLD fibrosis score (aORs = 1.88, 95% CI: 1.27 to 2.77) and FIB-4 index (aORs = 2.07, 95% CI: 1.24 to 3.45) yielded intermediate values, collectively demonstrating methodological heterogeneity without substantially altering the fundamental association. Geographical stratification identified Europe as exhibiting the strongest sarcopenia-fibrosis association (aORs = 2.36, 95% CI: 1.16 to 4.79), followed by North America (aORs = 2.13, 95% CI: 1.38 to 3.30) and Asia (aORs = 1.98, 95% CI: 1.40 to 2.80; Supplementary Table S7).

#### 3.4.6 Publication bias and sensitivity analysis

Among the studies of the aORs for NAFLD in patients with sarcopenia, visual inspection of the funnel plot revealed a symmetrical distribution (Supplementary Figure S5), and both the Egger test (*p* = 0.3) and Begg test (*p* = 0.033) were insignificant. Sensitivity analyses confirmed the robustness of NAFLD risk estimates, demonstrating a negligible influence of any single study (Supplementary Figure S6). In contrast, fibrosis studies exhibited measurable publication bias, as evidenced by funnel plot asymmetry (Supplementary Figure S7) and borderline significant Egger’s test (*p* = 0.041). Trim-and-fill imputation identified five missing studies, with the adjusted pooled estimate remaining statistically significant (aORs = 1.50, 95% CI: 1.10 to 2.06; Supplementary Figure S8). The preserved effect magnitude post-adjustment, coupled with stable sensitivity analyses (Supplementary Figure S9), substantiates the reliability of our fibrosis findings despite detectable bias.

## 4 Discussion

### 4.1 Summary of main results and mechanism basis

Through a synthesis of the latest evidence, this systematic review, employing stringent methodological safeguards (including the exclusion of overlapping datasets), revealed that sarcopenia is significantly associated with NAFLD progression and also linked to an increased risk of fibrotic evolution, thereby suggesting it as a potentially important risk factor for hepatic morbidity. Our findings confirm the established association between sarcopenia and increased NAFLD risk, consistent with prior meta-analyses. Through detailed subgroup stratification, this updated analysis reveals how methodological choices, particularly the use of non-comprehensive diagnostic criteria and different assessment modalities, critically influence prevalence estimates and risk associations. These insights extend beyond confirming the relationship by clarifying key sources of heterogeneity and underscoring the need for standardized approaches in future research.

NAFLD and sarcopenia, two increasingly prevalent age-related conditions, have overlapping etiologies, rooted in physical inactivity and malnutrition. Beyond these shared risk factors, comorbidities arise from intertwined pathophysiological mechanisms, including insulin resistance (IR), chronic low-grade inflammation, dysregulation of muscle-liver crosstalk mediators, hormonal imbalances (e.g., sex steroids and growth hormone/IGF-1 axis), and gut microbiota alterations (10, 11).

IR serves as the central pathological nexus that links sarcopenia and NAFLD progression by orchestrating a self-perpetuating metabolic dysfunction cycle. In adipose tissue, IR-driven lipolysis floods circulation with free fatty acids that accumulate as ectopic lipids in the muscle and liver, simultaneously inhibiting *β*-oxidation through hyperinsulinemia (57, 58). Studies have shown that patients with sarcopenia exhibit metabolic disorders in skeletal muscle, including impaired branched-chain amino acids (BCAAs) catabolism and mitochondrial dysfunction. BCAAs overload aberrantly stimulates mTOR signaling, aggravating IR via PI3K-Akt pathway suppression and oxidative phosphorylation deficits, ultimately accelerating proteolysis and muscle atrophy (3). Additionally, reduced activity of the PI3K/Akt signaling pathway leads to the accumulation of FoxO transcription factors and induces the upregulation of atrogen-1 and MuRF1, exacerbating skeletal muscle atrophy and NAFLD (59). Inflammation and oxidative stress bidirectionally regulate sarcopenia and NAFLD through the liver-muscle axis. Hepatic lipid accumulation triggers Toll-like receptor 4-dependent Kupffer cell activation, releasing IL-6 and TNF-*α*, which promotes liver fibrosis via TGF-*β* and induces muscle wasting through NF-κB-mediated ubiquitin-proteasome system activation (60, 61). This inflammatory milieu is exacerbated by sarcopenia-related mitochondrial dysfunction, which generates reactive oxygen species that further impair hepatic function through myokine imbalance (62). The pathogenic cascade culminates in hyperammonemia-induced NF-κB-p65 activation, which amplifies muscle atrophy via myostatin overexpression, whereas stellate cell-derived alpha-smooth muscle actin promotes progressive liver fibrosis (63). This dysregulation of the muscle-liver axis is further reflected at the molecular level by the endocrine dysfunction of skeletal muscle. On one hand, the secretion of protective myokines is diminished. For instance, exercise-induced irisin activates the hepatic AMPK pathway, promoting lipid clearance and improving mitochondrial function; its serum levels are decreased in NAFLD patients and inversely correlate with intrahepatic lipid deposition (64, 65). On the other hand, pathological myokine signaling is enhanced. Myostatin, for example, not only inhibits muscle protein synthesis and exacerbates atrophy but also directly activates hepatic stellate cells, driving fibrogenesis (10). Crucially, the systemic low-grade inflammatory state triggered by hepatic inflammation (involving IL-6, TNF-*α*, etc.) reciprocally affects skeletal muscle, transforming it from an inflammatory “target organ” into a persistent “contributor.” Under these conditions, muscle itself continuously secretes inflammatory factors such as IL-6, establishing a self-amplifying loop. This muscle-derived, sustained IL-6 signaling not only exacerbates hepatic insulin resistance and *de novo* lipogenesis via the JAK/STAT3 pathway (66) but also locally disrupts the muscle regenerative niche and impairs satellite cell function, thereby directly accelerating muscle loss (67, 68). Consequently, sarcopenia represents not merely a quantitative deficit in muscle mass but a decompensation of its endocrine and inflammatory regulatory functions. This dysfunction intertwines with hepatic pathology, jointly propelling disease progression toward fibrosis.

Regulation of skeletal muscle growth and development involves a complex interplay of various hormonal signals. As a key anabolic hormone, testosterone plays a crucial role in preserving muscular integrity and function (69, 70). Mechanistic studies have revealed its dual role in stimulating satellite cell proliferation and potentiating calcium-mediated anabolic signaling through membrane receptor activation (71). Clinical observations indicate that testosterone deficiency is a significant risk factor for age-related muscle loss (72), whereas replacement therapies are effective in counteracting muscle wasting. Notably, research has identified an independent correlation between low circulating testosterone levels and both the incidence and progression of nonalcoholic steatohepatitis in male patients (73). The GH/IGF-1 axis, another critical anabolic pathway, undergoes a progressive decline with age. Studies have reported that patients with NAFLD exhibit lower IGF-1 levels than healthy individuals (74), whereas recombinant human GH administration reduces hepatic steatosis in obese adolescents (75), highlighting the therapeutic potential of endocrine modulation. Adipose-derived adiponectin modulates lipid metabolism through insulin signaling pathways following receptor binding (76). This adipokine also exhibits anti-inflammatory properties by counteracting TNF-*α* activity, thereby ameliorating hepatic steatosis and inflammatory responses (77). Vitamin D modulates calcium phosphate homeostasis, and upon activation of the vitamin D receptor, exerts essential regulatory effects on skeletal integrity, muscular function, and hepatic metabolic processes. Inadequate vitamin D status disrupts muscle protein dynamics, diminishes contractile capacity, and predisposes patients to functional impairment (78). Moreover, vitamin D insufficiency may aggravate NAFLD progression through profibrotic and inflammatory mechanisms (79), whereas vitamin D supplementation improves both muscle function and hepatic insulin sensitivity (80, 81). Bidirectional communication networks between the intestine, liver, and skeletal muscle establish a foundation for microbial and metabolic crosstalk (82). The intestinal microbiota and its metabolites play a critical role in the development of both NAFLD and sarcopenia (83). Characteristic dysbiosis patterns emerge in sarcopenic individuals, marked by the depletion of *Bifidobacterium adolescentis* and *Prevotella copri*, microbial species whose metabolite nicotinic acid enhances mitochondrial bioenergetics in skeletal muscles (84, 85). Preclinical models have demonstrated that *Lactobacillus casei* Shirota supplementation improves muscle mass and strength in aging subjects, whereas cirrhotic patients exhibit distinct sarcopenia-associated microbiota signatures (86). Notably, prebiotic interventions modulate microbial ecology to improve hepatic lipid metabolism and reduce steatosis, suggesting microbiota-targeted approaches may concurrently address both conditions.

This study suggests a bidirectional association between sarcopenia and NAFLD with stage-dependent predominant drivers. In early stages, sarcopenia may promote steatosis by exacerbating insulin resistance and lipid spillover; once NAFLD progresses to significant fibrosis, hepatic inflammation and secretory dysfunction (e.g., myostatin release) become key drivers of muscle loss, forming a self-reinforcing pathological cycle (10, 49, 87). Thus, their relationship represents a dynamically evolving bidirectional vicious cycle rather than unidirectional causality (54, 88).

### 4.2 Subgroup analysis of different methods of sarcopenia measurement

In a meta-analysis of sarcopenia prevalence conducted in subgroups according to adjustment methods of SMI, diagnostic criteria of sarcopenia, and study regions, the differences between the subgroups were significant. Our stratified meta-analysis revealed a significant variation in sarcopenia prevalence among patients with NAFLD depending on the assessment methodology. SMI_wt yielded an approximately three-fold higher prevalence than SMI_ht^2^, with SMI_CT showing concordance with SMI_wt and SMI_BMI, producing intermediate values. This pattern persisted despite the increasing adoption of weight-normalized approaches (e.g., appendicular lean mass/weight×100) in recent research. When analyzed continuously, class I (−1 to −2 SD below young adult reference) and class II (< −2 SD) sarcopenia demonstrated expected prevalence gradients (13, 89).

Non-comprehensive (muscle mass-only) criteria inflated sarcopenia prevalence by 400% compared to comprehensive assessments (26% vs. 6%, *p* < 0.001), particularly when SMI_wt was used. This discrepancy persisted across studies despite the current guidelines that emphasize muscle strength as the core diagnostic criterion (1, 4). Intriguingly, studies employing multiple modalities consistently found that SMI_wt yielded a higher prevalence than SMI_BMI or SMI_ht^2^ (14, 34, 46, 62). Asian populations showed a significantly greater prevalence than European/South American cohorts, whereas North American estimates showed non-significant elevation. Subgroup analyses revealed no significant variations in study design, measurement techniques, or NAFLD diagnostic methods.

However, subgroup analysis of the pooled aORs for NAFLD and liver fibrosis revealed no significant differences across multiple strata, including the study design, muscle mass measurement techniques, SMI adjustment methods, sarcopenia diagnostic criteria, NAFLD assessment approaches, demographic characteristics (sex and age), or geographical regions. The aORs for NAFLD association remained remarkably consistent across all muscle assessment modalities (BIA, DXA, and CT) and SMI adjustment approaches (SMI_wt, SMI_ Ht^2^, and SMI_BMI). In contrast, fibrosis risk estimates demonstrated methodological dependence, with BIA-based assessments yielding significantly higher aORs than DXA, likely attributable to BIA’s practical advantages of BIA as a noninvasive, cost-effective bedside tool, particularly suited for serial measurements in hepatology practice.

### 4.3 Subgroup analysis of different methods of NAFLD diagnosis

In prevalence studies, NAFLD cases diagnosed by liver biopsy consistently demonstrated the highest incidence of sarcopenia compared to those identified through abdominal ultrasonography, transient elastography, or other diagnostic modalities (including CT, MRI, and fatty liver index). This pattern likely reflects the superior diagnostic reliability of liver biopsy as the reference standard despite the absence of statistically significant differences in prevalence rates between diagnostic methods. Notably, the strength of the association between sarcopenia and NAFLD varied according to the diagnostic approach. Although abdominal US was the most frequently used modality in the included studies, CT-diagnosed NAFLD cases exhibited the most pronounced aORs. The higher aORs observed with CT likely reflects its ability to concurrently assess body composition, including visceral fat and muscle mass, thus capturing metabolic dysregulation common to both conditions (1). In contrast, TE excels in detecting fibrosis but may be less sensitive to steatosis-driven metabolic alterations that precede structural liver damage. The association magnitudes for US, liver biopsy, and alternative diagnostic methods remained comparable with intergroup differences failing to reach statistical significance.

Transient elastography demonstrated exceptional discriminative capacity in liver fibrosis stratification analysis, revealing a 2.5-fold increased risk of sarcopenia compared with other modalities. Furthermore, sarcopenia was more strongly associated with significant fibrosis than with advanced fibrosis stages. These findings suggest that transient elastography is the preferred non-invasive screening tool for fibrosis-related sarcopenia risk assessment in both research and clinical settings.

### 4.4 Reasons for publication bias

Most of the included studies demonstrated robust methodological quality with a low risk of bias. Notably, while publication bias was evident among investigations examining the association between sarcopenia and NAFLD-related liver fibrosis, the aORs retained statistical significance even after imputation of five hypothetical studies via the trim-and-fill method. Nevertheless, the current body of literature addressing the influence of sarcopenia on fibrosis progression remains limited. Furthermore, the pathophysiological interplay between sarcopenia and NAFLD, wherein disease progression exacerbates hepatic fibrosis, appears to amplify the risk of fibrosis attributable to sarcopenia beyond that observed in NAFLD alone. This mechanistic synergy may underlie the observed publication bias in fibrosis-focused studies.

### 4.5 Reasons for the heterogeneity

Despite rigorous subgroup and sensitivity analyses, substantial heterogeneity persisted across our meta-analysis, which may be attributed to four key methodological considerations. The diagnostic approach for sarcopenia predominantly relies on the LMM criteria, with only a minority of studies employing comprehensive diagnostic standards. The applied cut-off values for LMM and LMS were derived from disparate guideline consensus documents. Second, although most investigations have incorporated multiple SMI adjustment methodologies, considerable variability in sarcopenia incidence estimates persisted. Furthermore, divergent NAFLD severity and hepatic fibrosis stages among the study populations coupled with heterogeneous diagnostic modalities likely contributed to inconsistent risk estimations. Finally, the geographic distribution of the included studies exhibited notable skewness, with a predominant representation from Asian populations, potentially limiting the generalizability. Meta-regression confirmed that SMI adjustment method and sarcopenia diagnostic criteria were significant sources of heterogeneity for NAFLD prevalence (Supplementary Table S8), aligning with our subgroup findings. However, for NAFLD risk, none of the tested covariates, including age, BMI, and methodological factors, significantly explained the variability, suggesting that unmeasured clinical differences such as comorbidities may account for the remaining heterogeneity.

### 4.6 Translational medicine perspective

Aberrant BCAAs catabolism may link sarcopenia with NAFLD progression (3); dietary BCAAs restriction trials are warranted. Testosterone and IGF-1 deficiency represent readily measurable screening indicators for high-risk male NAFLD populations (73).

### 4.7 Strengths and limitations

This rigorous meta-analysis provides precise prevalence estimates for sarcopenia in NAFLD populations by employing systematic search strategies and quality assessments to ensure a comprehensive evidence synthesis. However, several limitations include cross-sectional design precluding causal inference, and the limited number of longitudinal cohort studies further restricts our ability to delineate temporal sequence and establish causal direction between sarcopenia and NAFLD progression, significant heterogeneity in muscle assessment methods, particularly mass-only approaches inflating prevalence, and a lack of African data affecting generalizability. Furthermore, the majority of included studies were conducted in Asian populations, which may limit the extrapolation of our findings to other ethnic groups. Additionally, the accuracy limitations of ultrasound diagnosis of NAFLD may underestimate the true association strength; future validation via MRI-PDFF or biopsy is needed. Finally, publication bias tests and the trim-and-fill adjustment are underpowered in small meta-analyses like our fibrosis analysis (8 studies), limiting their reliability. Future studies should standardize diagnostic criteria, include diverse populations, and employ advanced analytical approaches; in particular, adopting comprehensive definitions would mitigate the four-fold over-diagnosis observed with mass-only cut-offs (Supplementary Table S6). In light of the ongoing nomenclature shift from NAFLD to MASLD, we maintained “NAFLD” throughout this manuscript for consistency with the included studies. Future meta-analyses are encouraged to follow updated MASLD diagnostic criteria once widely adopted.

## 5 Conclusion

This meta-analysis reveals that sarcopenia is both highly prevalent and strongly linked to NAFLD and its fibrotic progression, establishing muscle health as a critical and modifiable target in NAFLD management. We recommend routine primary-care screening using SARC-F combined with low-cost BIA, adoption of comprehensive diagnostic criteria to reduce overdiagnosis, and implementation of exercise-based and nutritional interventions to improve muscle mass and liver health. Large-scale prospective trials are warranted to validate this multifaceted strategy in NAFLD cohorts.

## Data Availability

The original contributions presented in the study are included in the article/Supplementary material, further inquiries can be directed to the corresponding author.
