# Supplementary material for: Sarcopenia as a risk factor for nonalcoholic fatty liver disease and liver fibrosis: an updated systematic review and meta-analysis
Source: Front Nutr. 2026 Mar 9;13:1726600. doi: 10.3389/fnut.2026.1726600 (PMC13006315; doi:10.3389/fnut.2026.1726600)
Supplement: Supplementary file 1 [file Data_Sheet_1.pdf]

Supplementary Material

1 Supplementary Tables and Figures

1.1 Supplementary Tables

Table S1 Search strategy by Pubmed, Embase, Medline, Cochrane library and Web of Science

1. sarcopenia'/exp
2. 'sarcopenia':ab,ti OR sarcopenias:ab,ti OR sarcopenic:ab,ti OR myopenia:ab,ti OR dynapenia:ab,ti OR 'muscle atrophy':ab,ti OR 'muscle wasting':ab,ti OR 'muscle loss':ab,ti OR 'muscle weak':ab,ti OR 'muscle decline':ab,ti OR 'muscular atrophy':ab,ti OR 'muscular wasting':ab,ti OR 'muscular loss':ab,ti OR 'muscular decline':ab,ti OR 'muscle mass':ab,ti OR 'skeletal muscle index':ab,ti OR smi:ab,ti OR 'muscle strength':ab,ti OR 'hand strength':ab,ti OR 'grip strength':ab,ti OR 'walking speed*':ab,ti OR 'speed*, walking':ab,ti OR 'walking pace*':ab,ti OR 'pace*, walking':ab,ti OR 'gait speed*':ab,ti OR 'speed*, gait':ab,ti
3. 'body composition'/exp
4. 'body composition*':ab,ti OR 'composition*, body':ab,ti
5. #1 OR #2 OR #3 OR #4
6. 'nonalcoholic fatty liver'/exp
7. 'non alcoholic fatty liver disease':ab,ti OR 'nonalcoholic fatty liver disease':ab,ti OR 'fatty liver, nonalcoholic':ab,ti OR 'fatty livers, nonalcoholic':ab,ti OR 'liver, nonalcoholic fatty':ab,ti OR 'livers, nonalcoholic fatty':ab,ti OR 'nonalcoholic fatty liver':ab,ti OR 'nonalcoholic fatty livers':ab,ti OR 'nonalcoholic steatohepatitis':ab,ti OR 'nonalcoholic steatohepatitides':ab,ti OR 'steatohepatitides, nonalcoholic':ab,ti OR 'steatohepatitis, nonalcoholic':ab,ti OR 'non-alcoholic steatohapatitis':ab,ti OR 'non alcoholic steatohapatitis':ab,ti OR 'non-alcoholic fatty liver disease':ab,ti OR 'non-alcoholic fatty liver':ab,ti OR 'fatty liver':ab,ti OR 'metabolic dysfunction-associated fatty liver disease':ab,ti OR 'metabolic dysfunction-associated steatotic liver disease':ab,ti OR 'metabolic associated fatty liver disease':ab,ti OR 'nash':ab,ti OR 'nafld':ab,ti OR 'mafld':ab,ti OR 'masld':ab,ti
8. #6 OR #7
9. 'prevalence'/exp
10. 'prevalence*':ab,ti OR 'point prevalence*':ab,ti
11. 'incidence*'
12. 'incidence*':ab,ti OR 'incidence proportion*':ab,ti OR 'incidence rate':ab,ti OR 'occurrence rate':ab,ti OR 'event rate':ab,ti OR frequency:ab,ti
13. 'odds ratio'/exp
14. 'odds ratio*':ab,ti OR 'relative odds':ab,ti OR 'odds, relative':ab,ti OR 'risk ratio*':ab,ti OR 'ratio*, risk':ab,ti OR 'or':ab,ti OR 'rr':ab,ti
15. 'risk'/exp
16. 'risk*':ab,ti OR 'relative risk*':ab,ti OR 'risk*, relative':ab,ti OR 'hazard ratio':ab,ti OR hr:ab,ti

17. #9 OR #10 OR #11 OR #12 OR #13 OR #14 OR #15 OR #16
18. #5 AND #8 AND #17

Table S2 Confounder adjustment of odds ratios for nonalcoholic fatty liver disease or liver fibrosis

First author and year	Confounder adjustment of NAFLD	Confounder adjustment of fibrosis
Hong (2014)	age, sex, smoking status, physical activity, homeostasis model of insulin resistance (HOMA-IR), high sensitivity C-reactive protein (hsCRP), and 25-hydroxyvitamin D (25[OH]D) levels.	/
Hashimoto_M (2016)	age, body mass index (BMI), smoking status, triglycerides (TG)/ high-density lipoprotein cholesterol (HDL-C) ratio, hemoglobin A1c (HbA1c) and gamma-glutamyl transferase.	/
Hashimoto_W (2016)	age, BMI, smoking status, TG / HDL-C ratio, HbA1c and gamma-glutamyl transferase.	/
Kim_M (2016)	age, smoking status (nonsmoker/past smoker/current smoker), alcohol drinking status (nondrinker/<20 g/day for males or <10 g/day for females), regular exercise (no/yes), white blood cell (WBC), HOMA-IR, 25[OH]D levels, number of metabolic syndrome, diabetes, and hypertension	/
Kim_W (2016)	age, smoking status (nonsmoker/past smoker/current smoker), alcohol drinking status (nondrinker/<20 g/day for males or	/

	<10 g/day for females), regular exercise (no/yes), total energy intake, carbohydrate intake (energy %), and fat intake (energy %), WBC, HOMA-IR, 25[OH]D levels, number of metabolic syndrome, diabetes, and hypertension	
Hong_wt (2020)	age, gender, forced vital capacity, hypertension, diabetes mellitus, and metabolic syndrome	/
Hong_BMI (2020)	age, gender, forced vital capacity, hypertension, diabetes mellitus, and metabolic syndrome	/
Hong_ht^2 (2020)	age, gender, forced vital capacity, hypertension, diabetes mellitus, and metabolic syndrome	/
Song (2022)	age, sex, systolic blood pressure (SBP), fasting plasma glucose (FBP), presence of diabetes, hypertension, smoking, and exercise	age, sex, SBP, FBP, presence of hypertension, smoking, and exercise
Kim_M (2023)	age, regular aerobic exercise, regular resistance exercise, visceral fat obesity (VFO), diabetes, hsCRP, and sarcopenia or myosteatosis	/
Kim_W (2023)	age, regular aerobic exercise, regular resistance exercise, VFO, diabetes, hsCRP, menopausal status, and sarcopenia or myosteatosis	/
Zhang (2022)	age, gender, SBP, diastolic blood pressure (DBP), HOMA-IR, TG, HDL-c, low-density lipoprotein	/

	cholesterol (LDL-C), BMI, waist circumference (WC), diabetes durations and smoking status.	
Lee_GSHC (2021)	age, sex, physical activity, smoking status, current drinking status, basal energy expenditure, mean blood pressure, fasting glucose, total cholesterol (TC), and history of cardiovascular disease (CVD).	/
Lee_KNHANES (2021)	age, sex, physical activity, smoking status, current drinking status, basal energy expenditure, daily protein intake, mean blood pressure, fasting glucose, TC, and history of CVD.	/
Lee_koGES (2021)	age, sex, physical activity, smoking status, current drinking status, basal energy expenditure, daily protein intake, mean blood pressure, fasting glucose, TC, and history of CVD.	/
Seo_M_wt (2019)	age, BMI, WC, SBP, DBP, HbA1c, TG (log scale), HDL-C, use of sulphonyl urea, thiazolidinedione, insulin, hsCRP, short insulin tolerance test.	/
Seo_W_wt (2019)	age, BMI, WC, SBP, DBP, HbA1c, TG (log scale), HDL-C use of sulphonyl urea, thiazolidinedione, insulin, hsCRP, short insulin tolerance test.	/
Seo_M_BMI (2019)	age, BMI, WC, SBP, DBP, HbA1c, TG (log scale), HDL-C, use of sulphonyl urea, thiazolidinedione,	/

	insulin, hsCRP, short insulin tolerance test.	
Seo_W_BMI (2019)	age, BMI, WC, SBP, DBP, HbA1c, TG (log scale), HDL-C, use of sulphonyl urea, thiazolidinedione, insulin, hsCRP, short insulin tolerance test.	/
Wang_M (2021)	age, weight, BMI, TG, alanine aminotransferase (ALT).	/
Wang_W (2021)	age, weight, BMI, TG, ALT.	/
Wijarnpreecha (2019)	age, sex, ethnicity, BMI, economic status, diabetes, smoking status, hypertension, TC, anti-hyperlipidemia medication, sedentary physical activity, vitamin D deficiency, and C-reactive protein.	age, gender, ethnicity, WC, diabetes, smoking status, hypertension, TC, sedentary physical activity, vitamin D deficiency, and C-reactive protein.
Choe (2018)	age, sex, WC, SBP, fasting glucose, TG, HDL-C, and smoking status.	/
Gan (2020)	age, gender, residence area, smoking, physical activity, height, ALT, TG, LDL-c, TC, HbA1c, uric acid (UA), hypertension, diabetes, hsCRP level, HOMA-IR, and current medications.	/
Kang (2020)	age, gender, obesity, diabetes, hypertension, hyperuricemia, serum TG, high-density lipoprotein, C-reactive protein, history of small bowel resection, moderate to severe inflammatory bowel disease (IBD),	/

	duration of IBD, use of steroid, azathioprine, biologics, total parenteral nutrition, number of hospitalizations	
Park (2020)	age, height, weight, sex (male), aspartate aminotransferase (AST), ALT, fasting glucose, TG, and WC.	/
Jiang_M (2021)	Age, duration of type 2 diabetes mellitus, BMI, WC, WBC count, estimated glomerular filtration rate, hemoglobin, TG, HDL-C, ALT, serum UA, use of anti-diabetic drugs.	/
Jiang_W (2021)	Age, duration of type 2 diabetes mellitus, BMI, WC, WBC count, estimated glomerular filtration rate, hemoglobin, TG, HDL-C, ALT, serum UA, use of anti-diabetic drugs.	/
Afferink_M (2019)	age, study cohorts, weight, height, HOMA-IR, TG, and android-to-gynoid fat ratio (AGR).	/
Afferink_W (2019)	age, study cohorts, weight, height, HOMA-IR, triglycerides, and AGR.	/
Chung (2019)	age, sex, visceral fat area, hypertension and diabetes mellitus, TC, and LDL-C	/
Chung (2023)	Age, sex, hypertension, diabetes mellitus, dyslipidemia, smoking, and C-reactive protein levels	Age $\geq$ 50 years, sex, hypertension, diabetes mellitus, dyslipidemia, smoking, and C-reactive protein levels
Pan (2022)	age, BMI, serum creatinine levels,	age, BMI, serum creatinine

	and diabetes	levels, and diabetes
Guo (2022)	age, sex, BMI, SBP, DBP, FPG, HbA1c, TC, TG, LDL-C, HDL-C and UA	age, sex, BMI, SBP, DBP, FPG, TG, HDL-C and UA
Kang_wt (2019)	/	sex, FBP, presence of hypertension, presence of obesity, TC, TG, high-density lipoprotein, and hsCRP.
Kang_BMI (2019)	/	sex, FBP, presence of hypertension, presence of obesity, TC, TG, high-density lipoprotein, and hsCRP.
Petta (2016)	/	demographic, metabolic and histological confounders
Zhu (2023)	/	age and sex
Zhang_NHANES (2025)	/	gender, age, smoke, alcohol, physical activity, diabetes mellitus, hypertension
Zhang (2025)	/	gender, age, smoke, alcohol, physical activity, diabetes mellitus, hypertension

Abbreviations: NAFLD, nonalcoholic fatty liver disease; wt: weight; ht^2: height square.

Table S3 The reasons for the exclusion of full-text articles

Study	Reason for the exclusion
Kwon Y(1) (2020)	Exclusion reason: participants under 18 years
Han E(2) (2020)	This article applied the same dataset with an included study/ No available aORs values for sarcopenia's effect on NAFLD or hepatic fibrosis.
Yang HJ(3) (2023)	Not reported sarcopenia prevalence data in NAFLD patients/No available aORs values for sarcopenia's effect on NAFLD or hepatic fibrosis.
Zhang S(4) (2023)	Exclusion study types: review
Jung HN(5) (2023)	Exclusion study types: review
Lee YH(6) (2016)	This article applied the same dataset with an included study.
Pacifico L(7) (2020)	Exclusion reason: participants under 18 years.
Moon JS(8) (2013)	Not reported sarcopenia prevalence data in NAFLD patients/No available aORs values for sarcopenia's effect on NAFLD or hepatic fibrosis.
Hong J(9) (2021)	This article applied the same dataset with an included study/No available aORs values for sarcopenia's effect on NAFLD or hepatic fibrosis.
Shen H(10) (2016)	Exclusion study types: conference abstract
Choi YJ(11) (2014)	Exclusion study types: conference abstract
Kumar A(12) (2025)	Not reported sarcopenia prevalence data in NAFLD patients/No available aORs values for sarcopenia's effect on NAFLD or hepatic fibrosis.
Yang H(13) (2024)	Not reported sarcopenia prevalence data in NAFLD patients/No available aORs values for sarcopenia's effect on NAFLD or hepatic fibrosis.
Cheng Z(14) (2024)	Not reported sarcopenia prevalence data in NAFLD patients/No available aORs values for sarcopenia's effect on NAFLD or hepatic fibrosis.
Wan Q(15) (2024)	Not reported sarcopenia prevalence data in NAFLD patients/No available aORs values for sarcopenia's effect on NAFLD or hepatic fibrosis.

Bhatia SJ(16) (2023)	Exclusion study types: editorial
Szczepanek-Parulska E(17) (2022)	Exclusion study types: review
Ushiro K(18) (2024)	Not reported sarcopenia prevalence data in NAFLD patients/No available aORs values for sarcopenia's effect on NAFLD or hepatic fibrosis.
Ushiro K(19) (2024)	Not reported sarcopenia prevalence data in NAFLD patients/No available aORs values for sarcopenia's effect on NAFLD or hepatic fibrosis.
Tobaruela-Resola AL(20) (2024)	Not reported sarcopenia prevalence data in NAFLD patients/No available aORs values for sarcopenia's effect on NAFLD or hepatic fibrosis.
Sheptulina AF(21) (2024)	Exclusion study types: review
Onishi S(22) (2024)	Not reported sarcopenia prevalence data in NAFLD patients/No available aORs values for sarcopenia's effect on NAFLD or hepatic fibrosis.
Boutari C(23) (2024)	Not reported sarcopenia prevalence data in NAFLD patients/No available aORs values for sarcopenia's effect on NAFLD or hepatic fibrosis.
Polyzos SA(24) (2023)	Exclusion study types: review
Onishi S(25) (2023)	Not reported sarcopenia prevalence data in NAFLD patients/No available aORs values for sarcopenia's effect on NAFLD or hepatic fibrosis.
Kawanaka M(26) (2023)	Not reported sarcopenia prevalence data in NAFLD patients/No available aORs values for sarcopenia's effect on NAFLD or hepatic fibrosis.
Di Ciaula A(27) (2023)	Exclusion study types: comment
Shida T(28) (2018)	Not reported sarcopenia prevalence data in NAFLD patients/No available aORs values for sarcopenia's effect on NAFLD or hepatic fibrosis.
Kim LJ(29) (2011)	Not reported sarcopenia prevalence data in NAFLD patients/No available aORs values for sarcopenia's effect on NAFLD or hepatic fibrosis.
Onitsuka Y(30) (2014)	Not reported sarcopenia prevalence data in NAFLD patients/No available aORs values for sarcopenia's effect on NAFLD or hepatic fibrosis.
Vassilatou E(31)	Exclusion study types: review

(2014)	
Koch M(32) (2015)	Not reported sarcopenia prevalence data in NAFLD patients/No available aORs values for sarcopenia's effect on NAFLD or hepatic fibrosis.
Kim JY(33) (2016)	Not reported sarcopenia prevalence data in NAFLD patients/No available aORs values for sarcopenia's effect on NAFLD or hepatic fibrosis.
Stankevicius C(34) (2024)	Exclusion study types: review
Mantovani A(35) (2020)	Exclusion study types: review
Radmard AR(36) (2016)	Not reported sarcopenia prevalence data in NAFLD patients/No available aORs values for sarcopenia's effect on NAFLD or hepatic fibrosis.
Yen CH(37) (2017)	Not reported sarcopenia prevalence data in NAFLD patients/No available aORs values for sarcopenia's effect on NAFLD or hepatic fibrosis.
Yamamoto R(38) (2023)	Not reported sarcopenia prevalence data in NAFLD patients/No available aORs values for sarcopenia's effect on NAFLD or hepatic fibrosis.
Delzenne NM(39) (2022)	Exclusion study types: editorial
Singeap AM(40) (2021)	Exclusion study types: review

Abbreviations: NAFLD, nonalcoholic fatty liver disease; aORs, adjusted odds radios.

Table S4 Risk of bias of the included studies using the National Institutes of Health Quality Assessment Tool for Observational Cohort and Cross-Sectional Studies

First author (year)	Q1	Q2	Q3	Q4	Q5	Q6	Q7	Q8	Q9	Q10	Q11	Q12	Q13	Q14
Hong (2014)	Yes	Yes	Yes	Yes	No	No	Yes	Yes	Yes	NA	Yes	Yes	NA	Yes
Petta (2016)	Yes	Yes	Yes	Yes	No	No	Yes	NA	Yes	NA	Yes	Yes	NA	Yes
Park (2020)	Yes	Yes	Yes	Yes	No	No	Yes	NA	Yes	NA	Yes	NA	NA	Yes
Linge (2020)	Yes	Yes	Yes	Yes	No	No	No	Yes	Yes	NA	Yes	NA	NA	Yes
Song (2022)	Yes	Yes	Yes	Yes	No	No	Yes	Yes	Yes	NA	Yes	NA	NA	Yes
Kang (2020)	Yes	Yes	Yes	Yes	No	No	Yes	NA	Yes	NA	Yes	NA	NA	Yes
Gan (2020)	Yes	Yes	Yes	Yes	No	No	Yes	Yes	Yes	NA	Yes	NA	NA	Yes
Golabi (2020)	Yes	Yes	Yes	Yes	No	No	Yes	NA	Yes	NA	Yes	NA	NA	Yes
Choe (2018)	Yes	Yes	Yes	Yes	No	No	Yes	Yes	Yes	NA	Yes	Yes	NA	Yes
Wijarnpreecha (2019)	Yes	Yes	Yes	Yes	No	No	Yes	NA	Yes	NA	Yes	NA	NA	Yes
Kang (2019)	Yes	Yes	No	Yes	No	No	Yes	Yes	Yes	NA	Yes	NA	NA	Yes
Wang (2021)	Yes	Yes	Yes	Yes	No	No	Yes	Yes	Yes	NA	Yes	Yes	NA	Yes
Seo (2019)	Yes	Yes	No	Yes	No	No	Yes	Yes	Yes	NA	Yes	NA	NA	Yes
Lee (2021)	Yes	Yes	No	Yes	No	No	Yes	Yes	Yes	NA	Yes	NA	NA	Yes
Hashimoto (2016)	Yes	Yes	Yes	Yes	No	No	Yes	NA	Yes	NA	Yes	NA	NA	Yes
Hong (2020)	Yes	Yes	No	Yes	No	No	Yes	Yes	Yes	NA	Yes	NA	NA	Yes
Alferink (2019)	Yes	Yes	Yes	Yes	No	No	Yes	Yes	Yes	NA	Yes	NA	NA	Yes
Zhang (2022)	Yes	Yes	Yes	Yes	No	No	Yes	Yes	Yes	NA	Yes	Yes	NA	Yes
Chung (2019)	Yes	Yes	Yes	Yes	No	No	Yes	Yes	Yes	NA	Yes	NA	NA	Yes
Bhanji (2019)	Yes	Yes	Yes	Yes	No	Yes	No	NA	Yes	NA	Yes	Yes	NA	Yes
Kim (2016)	Yes	Yes	Yes	Yes	No	No	Yes	NA	Yes	NA	Yes	NA	NA	Yes
Debroy (2019)	Yes	Yes	Yes	Yes	No	No	No	Yes	Yes	NA	Yes	NA	NA	NR
Kim (2023)	Yes	Yes	Yes	Yes	No	No	Yes	Yes	Yes	NA	Yes	NA	NA	Yes
Zhu (2023)	Yes	Yes	Yes	Yes	No	No	Yes	NA	Yes	NA	Yes	NA	NA	Yes
Lee (2021)	Yes	Yes	Yes	Yes	No	No	Yes	Yes	Yes	NA	Yes	Yes	NA	Yes
Nachit (2021)	Yes	Yes	Yes	Yes	No	No	Yes	Yes	Yes	Yes	Yes	Yes	NA	NA

Almeida (2022)	Yes	Yes	Yes	Yes	No	No	No	Yes	Yes	NA	Yes	NA	NA	NA
Moon (2021)	Yes	Yes	Yes	Yes	No	No	No	Yes	Yes	NA	Yes	NA	NA	NA
Pan (2022)	Yes	Yes	Yes	Yes	No	Yes	Yes	Yes	Yes	NA	Yes	NA	NA	Yes
Chung (2023)	Yes	Yes	Yes	Yes	No	No	Yes	Yes	Yes	NA	Yes	NA	NA	Yes
Harring (2023)	Yes	Yes	No	Yes	No	No	Yes	Yes	Yes	NA	Yes	NA	NA	Yes
Kang (2021)	Yes	Yes	No	Yes	No	No	No	Yes	Yes	NA	Yes	NA	NA	NA
Jiang (2021)	Yes	Yes	Yes	Yes	No	No	Yes	Yes	Yes	NA	Yes	NA	NA	Yes
Guan (2022)	Yes	Yes	Yes	Yes	No	No	No	Yes	Yes	NA	Yes	NA	NA	NA
Cho (2023)	Yes	Yes	No	Yes	No	No	No	Yes	Yes	NA	Yes	NA	NA	NA
Seo (2022)	Yes	Yes	Yes	Yes	No	No	Yes	Yes	Yes	NA	Yes	NA	NA	NA
Guo (2022)	Yes	Yes	Yes	Yes	No	No	Yes	Yes	Yes	NA	Yes	NA	NA	Yes
Sheptulina (2023)	Yes	Yes	Yes	Yes	Yes	No	No	NA	Yes	NA	Yes	NA	NA	NA
Zhao (2023)	Yes	Yes	No	Yes	No	No	No	Yes	Yes	NA	Yes	NA	NA	Yes
Amer (2025)	Yes	Yes	Yes	Yes	Yes	Yes	No	NA	Yes	NA	Yes	NA	NA	NA
Zhang (2025)	Yes	Yes	No	Yes	No	No	Yes	Yes	Yes	NA	Yes	NA	NA	Yes

Yes = criteria met No = criteria not met NA = not applicable NR = not reported

Criteria

Q1. Was the research question or objective in this paper clearly stated?

Q2. Was the study population clearly specified and defined?

Q3. Was the participation rate of eligible persons at least 50%?

Q4. Were all the subjects selected or recruited from the same or similar populations (including the same time period)? Were inclusion and exclusion criteria for being in the study prespecified and applied uniformly to all participants?

Q5. Was a sample size justification, power description, or variance and effect estimates provided?

Q6. For the analyses in this paper, were the exposure(s) of interest measured prior to the outcome(s) being measured?

Q7. Was the timeframe sufficient so that one could reasonably expect to see an association between exposure and outcome if it existed?

Q8. For exposures that can vary in amount or level, did the study examine different levels of the exposure as related to the outcome (e.g., categories of exposure, or exposure measured as continuous variable)?

Q9. Were the exposure measures (independent variables) clearly defined, valid, reliable, and implemented consistently across all study participants?

Q10. Was the exposure(s) assessed more than once over time?

Q11. Were the outcome measures (dependent variables) clearly defined, valid, reliable, and implemented consistently across all study participants?
Q12. Were the outcome assessors blinded to the exposure status of participants?
Q13. Was loss to follow-up after baseline 20% or less?
Q14. Were key potential confounding variables measured and adjusted statistically for their impact on the relationship between exposure(s) and outcome(s)?

Table S5 Risk of bias of the included studies using assessment tool explicitly for prevalence studies

First author (year)	Q1	Q2	Q3	Q4	Q5	Q6	Q7	Q8	Q9	Q10	Overall
Petta (2016)	High	Low	High	High	Low	Low	Low	Low	Low	Low	Moderate
Park (2020)	High	Low	High	Low	Low	Low	Low	Low	Low	Low	Low
Linge (2020)	High	Low	High	High	Low	Low	Low	Low	Low	Low	Moderate
Kang (2020)	High	Low	High	Low	Low	Low	Low	Low	Low	Low	Low
Gan (2020)	Moderate	Low	High	High	Low	Low	Low	Low	Low	Low	Low
Golabi (2020)	Low	Low	Low	High	Low	Low	Low	Low	Low	Low	Low
Choe (2018)	High	Low	High	Low	Low	Low	Low	Low	Low	Low	Low
Kang (2019)	High	Low	High	Low	Low	Low	Low	Low	Low	Low	Low
Wang (2021)	High	Low	High	High	Low	Low	Low	Low	Low	Low	Moderate
Seo (2019)	High	Low	High	High	Low	Low	Low	Low	Low	Low	Moderate
Lee (2021)	Low	Low	Low	High	Low	Low	Low	High	Low	Low	Low
Alferink (2019)	Moderate	Low	High	High	Low	Low	Low	Low	Low	Low	Low
Zhang (2022)	High	Low	High	Low	Low	Low	Low	Low	Low	Low	Low
Chung (2019)	High	Low	High	Low	Low	Low	Low	Low	Low	Low	Low
Bhanji (2019)	High	Low	High	Low	Low	Low	Low	Low	Low	Low	Low
Debroy (2019)	High	Low	High	High	Low	Low	Low	Low	Low	Low	Moderate
Zhu (2023)	High	Low	High	High	Low	Low	Low	Low	Low	Low	Moderate
Lee (2021)	High	Low	High	High	Low	Low	Low	Low	Low	Low	Moderate
Nachit (2021)	High	High	High	Low	Low	Low	Low	Low	Low	Low	Moderate
Almeida (2022)	High	Low	High	High	Low	Low	Low	Low	Low	Low	Moderate
Moon (2021)	Low	Low	Low	High	Low	Low	Low	Low	Low	Low	Low
Pan (2022)	High	Low	High	Low	Low	Low	Low	Low	Low	Low	Low
Chung (2023)	Moderate	Low	High	Low	Low	Low	Low	Low	Low	Low	Low
Harring (2023)	Low	Low	Low	High	Low	Low	Low	Low	Low	Low	Low
Kang (2021)	High	Low	High	Low	Low	Low	Low	Low	Low	Low	Low
Jiang (2021)	High	Low	High	High	Low	Low	Low	Low	Low	Low	Moderate
Guan (2022)	High	Low	High	Low	Low	Low	Low	Low	Low	Low	Low
Cho (2023)	High	Low	High	Low	Low	Low	Low	Low	Low	Low	Low
Seo (2022)	High	Low	High	Low	Low	Low	Low	Low	Low	Low	Low

Sheptulina (2023)	High	Low	High	Low	Low	Low	Low	Low	Low	Low	Low
Zhao (2023)	Low	Low	Low	High	Low	Low	Low	Low	Low	Low	Low
Amer (2025)	High	Low	Low	Low	Low	Low	Low	Low	Low	Low	Low
Zhang (2025)	High	Low	High	Low	Low	Low	Low	Low	Low	Low	Low

Low = low risk of bias High = high risk of bias Moderate = moderate risk of bias

Criteria

External validity:

Q1. Was the study's target population a close representation of the national population in relation to relevant variables?

Q2. Was the sampling frame a true or close representation of the target population?

Q3. Was some form of random selection used to select the sample, OR was a census undertaken?

Q4. Was the likelihood of nonresponse bias minimal?

Internal validity:

Q5. Were data collected directly from the subjects (as opposed to a proxy)?

Q6. Was an acceptable case definition used in the study?

Q7. Was the study instrument that measured the parameter of interest shown to have validity and reliability?

Q8. Was the same mode of data collection used for all subjects?

Q9. Was the length of the shortest prevalence period for the parameter of interest appropriate?

Q10. Were the numerator(s) and denominator(s) for the parameter of interest appropriate?

Q11. Summary item on the overall risk of study bias.

Table S6 Stratified analyses between baseline characteristics and the prevalence of sarcopenia in nonalcoholic fatty liver disease

Subgroup	No.	Prevalence (95% CI)	P_h	I^2(%)	P_d
Study design					
Cross-sectional	41	0.24 (0.20, 0.27)	< 0.001	99.7	/
Cohort	5	0.18 (0.07, 0.29)	< 0.001	98.4	/
Subgroup total	46	0.23 (0.20, 0.26)	< 0.001	99.6	0.321
Muscle mass measure					
BIA	25	0.23 (0.18, 0.27)	< 0.001	99.7	/
DXA	16	0.24 (0.18, 0.29)	< 0.001	99.4	/
CT	4	0.26 (0.05, 0.48)	< 0.001	99.0	/
NA	1	0.16 (0.09, 0.23)	/	/	/
Subgroup total	46	0.23 (0.20, 0.26)	< 0.001	99.6	0.311
Adjustment of SMI					
SMI_wt	17	0.32 (0.23, 0.40)	< 0.001	99.8	/
SMI_ht^2	12	0.11 (0.08, 0.14)	< 0.001	97.9	/
SMI_BMI	12	0.21 (0.16, 0.27)	< 0.001	99.6	/
SMI_CT	4	0.26 (0.05, 0.48)	< 0.001	99.0	/
SMI_others	1	0.16 (0.09, 0.23)	/	/	/
Subgroup total	46	0.23 (0.20, 0.26)	< 0.001	99.6	< 0.001
Diagnosis criteria					
Comprehensive	7	0.06 (0.04, 0.09)	< 0.001	93.4	/
Non-comprehensive	39	0.26 (0.22, 0.29)	< 0.001	99.7	/
Subgroup total	46	0.23 (0.20, 0.26)	< 0.001	99.6	< 0.001
NAFLD diagnosis					
Abdominal US	32	0.23 (0.19, 0.27)	< 0.001	99.7	/
Transient elastography	4	0.24 (0.15, 0.32)	< 0.001	99.0	/
Liver biopsy	4	0.28 (0.12, 0.44)	< 0.001	99.4	/
Others	6	0.19 (0.11, 0.28)	< 0.001	99.3	/
Subgroup total	46	0.23 (0.20, 0.26)	< 0.001	99.6	0.764
Study region					
Asia	35	0.24 (0.21. 0.27)	< 0.001	99.4	/
Europe	6	0.09 (0.05, 0.12)	< 0.001	97.3	/
North America	4	0.33 (0.06, 0.60)	< 0.001	99.8	/
South America	1	0.04 (0.00, 0.08)	/	/	/
Subgroup total	46	0.23 (0.20, 0.26)	< 0.001	99.6	< 0.001
Sex					
Men	7	0.28 (0.11, 0.45)	< 0.001	99.2	/
Women	5	0.18 (0.02, 0.34)	< 0.001	99.2	/
Both	34	0.23 (0.19, 0.27)	< 0.001	99.7	/
Subgroup total	46	0.23 (0.20, 0.26)	< 0.001	99.6	0.715
Age					
< 60 years	30	0.22 (0.18, 0.27)	< 0.001	99.7	/
≥ 60 years	10	0.28 (0.14, 0.42)	< 0.001	99.3	/
NA	6	0.17 (0.10, 0.25)	< 0.001	99.1	/
Subgroup total	46	0.23 (0.20, 0.26)	< 0.001	99.6	0.312

Abbreviations: NAFLD, nonalcoholic fatty liver disease; BIA, bioelectrical impedance analysis; DXA, dual-energy X-ray absorptiometry; CT, Computed Tomography; abdominal US, abdominal ultrasound; SMI, skeletal muscle mass index; BMI, body mass index; wt, weight; ht^2, height square; No, number; P_h, P value for heterogeneity;

P_d, P value for difference; NA, not applicable.

Table S7 Stratified analyses between baseline characteristics and the risk for nonalcoholic fatty liver disease/liver fibrosis in sarcopenia patients

NAFLD subgroup	No.	aORs (95% CI)	P_h	I^2(%)	P_d	Fibrosis subgroup	No.	aORs (95% CI)	P_h	I^2(%)	P_d
Study design						Study design					
Cross-sectional	32	1.56 (1.34, 1.81)	< 0.001	91.3	/	Cross-sectional	10	2.04 (1.51, 2.75)	< 0.001	77.7	/
Cohort	2	2.47 (0.80, 7.66)	0.045	75.2	/	Cohort	1	2.07 (1.24, 3.45)	/	/	/
Subgroup total	34	1.58 (1.37, 1.82)	< 0.001	90.9	0.429	Subgroup total	11	2.03 (1.54, 2.68)	< 0.001	75.7	0.959
Muscle mass measure						Muscle mass measure					
BIA	16	1.53 (1.19, 1.96)	< 0.001	95.0	/	BIA	8	2.15 (1.48, 3.13)	< 0.001	77.6	/
DXA	14	1.67 (1.37, 2.03)	< 0.001	77.6	/	DXA	3	1.84 (1.12, 3.03)	0.02	74.4	/
CT	4	1.39 (1.25, 1.55)	0.266	24.1	/	Subgroup total	11	2.03 (1.54, 2.68)	< 0.001	75.7	0.624
Subgroup total	34	1.58 (1.37, 1.82)	< 0.001	90.9	0.263	Adjustment of SMI					
Adjustment of SMI						SMI_wt	7	2.06 (1.38, 3.07)	< 0.001	79.2	/
SMI_wt	19	1.66 (1.33, 2.09)	< 0.001	93.8	/	SMI_ht^2	1	2.07 (1.24, 3.45)	/	/	/
SMI_ht^2	6	1.67 (1.23, 2.28)	< 0.001	87.2	/	SMI_BMI	3	2.10 (1.08, 4.08)	0.006	80.4	/
SMI_BMI	5	1.33 (1.14, 1.54)	0.459	0	/	Subgroup total	11	2.03 (1.54, 2.68)	< 0.001	75.7	0.998
SMI_CT	4	1.39 (1.25, 1.55)	0.266	24.1	/	NAFLD diagnosis					
Subgroup total	34	1.58 (1.37, 1.82)	< 0.001	90.9	0.269	Abdominal US	7	2.30 (1.54, 3.42)	< 0.001	79.4	/
Diagnosis criteria						Transient elastography	2	1.80 (0.83, 3.93)	0.015	83.2	/
Comprehensive	5	1.70 (1.04, 2.79)	< 0.001	90.1	/	Liver biopsy	2	1.64 (0.88, 3.07)	0.158	49.9	/
Non-comprehensive	29	1.55 (1.34, 1.80)	< 0.001	91.2	/	Subgroup total	11	2.03 (1.54, 2.68)	< 0.001	75.7	0.636
Subgroup total	34	1.58 (1.37, 1.82)	< 0.001	90.9	0.724	Study region					
NAFLD diagnosis						Asia	8	1.98 (1.40, 2.80)	< 0.001	81.0	/
Abdominal US	22	1.64 (1.35, 2.00)	< 0.001	92.6	/	Europe	1	2.36 (1.16, 4.79)	1.0	0	/
Transient elastography	2	0.89 (0.74, 1.07)	0.161	49.2	/	North America	2	2.13 (1.38, 3.30)	0.22	33.5	/
CT	2	2.98 (1.39, 6.38)	0.225	32.1	/	Subgroup total	11	2.03 (1.54, 2.68)	< 0.001	75.7	0.901
Liver biopsy	1	1.65 (1.30, 2.10)	/	/	/	Sex					
Others	7	1.45 (1.30, 1.61)	0.203	29.4	/	Men	1	1.24 (0.72, 2.14)	/	/	/
Subgroup total	34	1.58 (1.37, 1.82)	< 0.001	90.9	< 0.001	Both	10	2.14 (1.59, 2.88)	< 0.001	77.4	/

Study region						Subgroup total	11	2.03 (1.54, 2.68)	< 0.001	75.7	0.083
Asia	31	1.60 (1.38, 1.86)	< 0.001	91.6	/	Age					
Europe	2	1.33 (0.58, 3.05)	0.11	60.8	/	< 60 years	10	2.04 (1.51, 2.75)	< 0.001	77.7	/
North America	1	1.24 (1.04, 1.48)	/	/	/	≥ 60 years	1	2.07 (1.24, 3.45)	/	/	/
Subgroup total	34	1.58 (1.37, 1.82)	< 0.001	90.9	0.093	Subgroup total	11	2.03 (1.54, 2.68)	< 0.001	75.7	0.959
Sex						Liver fibrosis diagnosis					
Men	9	1.35 (1.14, 1.59)	< 0.001	73.3	/	Transient elastography	4	2.49 (1.22, 5.05)	< 0.001	89.2	/
Women	8	1.21 (1.05, 1.39)	0.007	63.6	/	Liver biopsy	2	1.64 (0.88, 3.07)	0.158	49.9	/
Both	14	2.00 (1.49, 2.70)	< 0.001	94.7	/	NAFLD fibrosis score	4	1.88 (1.27, 2.77)	0.037	64.5	/
NA	3	1.61 (1.12, 2.31)	0.091	58.2		FIB-4 score	1	2.07 (1.24, 3.45)	/	/	/
Subgroup total	34	1.58 (1.37, 1.82)	< 0.001	90.9	0.018	Subgroup total	11	2.03 (1.54, 2.68)	< 0.001	75.7	0.842
Age						Liver fibrosis subgroup					
< 60 years	20	1.68 (1.40, 2.02)	< 0.001	93.4	/	Significant fibrosis	3	2.44 (1.02, 5.83)	0.004	81.9	/
≥ 60 years	9	1.28 (1.03, 1.60)	< 0.001	74.5	/	Advanced fibrosis	8	1.84 (1.44, 2.34)	0.013	60.7	/
NA	5	1.54 (1.14, 2.07)	0.117	45.8	/	Subgroup total	11	2.03 (1.54, 2.68)	< 0.001	75.7	0.534
Subgroup total	34	1.58 (1.37, 1.82)	< 0.001	90.9	0.176						

Abbreviations: NAFLD, nonalcoholic fatty liver disease; BIA: bioelectrical impedance analysis; DXA: dual-energy X-ray absorptiometry; CT: Computed Tomography; SMI, skeletal muscle mass index; wt: weight; ht^2: height square; BMI: body mass index; abdominal US, abdominal ultrasound; FIB-4 score: fibrosis 4 score; No: number; aORs: adjusted odds ratios; P_h: P value for heterogeneity; P_d: P value for difference; NA, not applicable.

Table S8 Meta-regression analysis of potential sources of heterogeneity for NAFLD prevalence and NAFLD risk (aORs) in sarcopenia patients

**NAFLD prevalence**	**No.**	P_h	I^2**_residual (%)**	**Adjusted** R^2 **(%)**	**NAFLD risk**	**No.**	P_h	I^2**_residual (%)**	**Adjusted** R^2 **(%)**
Sample size	46	0.124	99.63	3.10	Sample size	34	0.158	89.04	7.45
Age	40	0.444	99.67	-0.87	Age	29	0.144	91.91	5.10
BMI	39	0.836	99.65	-2.68	BMI	29	0.522	92.35	-2.74
Adjustment of SMI	46	0.039	99.63	7.36	Adjustment of SMI	34	0.939	90.85	-4.11
Diagnosis criteria	46	0.021	99.61	9.79	Diagnosis criteria	34	0.617	91.03	-1.60
Study region	46	0.590	99.62	-1.71	Study region	34	0.448	91.07	-2.54
Study design	46	0.467	99.64	-1.07	Study design	34	0.695	90.18	-1.32
Sex	46	0.677	99.64	-1.79	Sex	34	0.097	90.17	10.18
Muscle mass measure	46	0.681	99.64	-1.94	Muscle mass measure	34	0.516	91.16	-2.50
NAFLD diagnosis	46	0.661	99.64	-1.87	NAFLD diagnosis	34	0.271	90.83	1.70

Abbreviations: NAFLD, nonalcoholic fatty liver disease; BMI: body mass index; SMI, skeletal muscle mass index; No: number; aORs: adjusted odds ratios; Ph: P value for heterogeneity.

1.2 Supplementary Figures

Figure S1 The pooled estimated prevalence rates of sarcopenia in patients without nonalcoholic fatty liver disease. CI, confidence interval

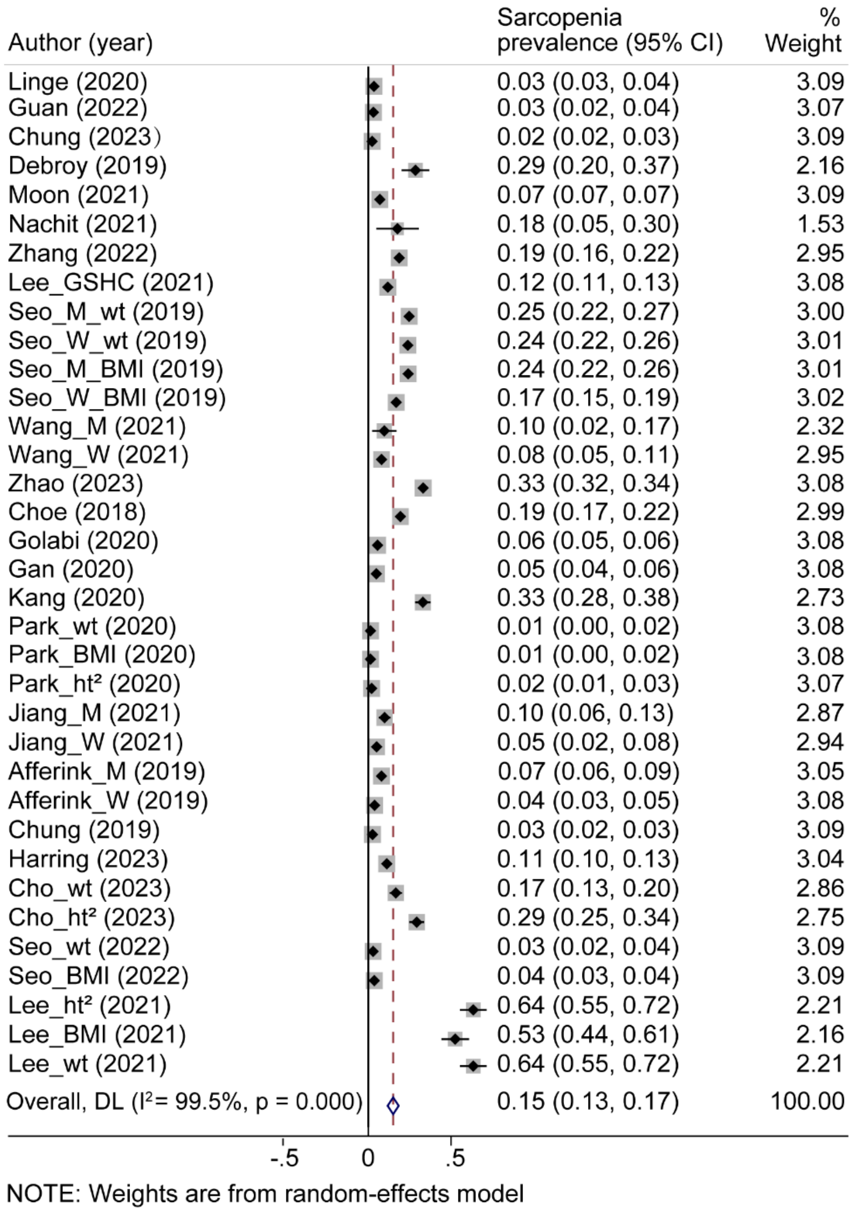

Figure S2 Funnel plot for publication bias assessment in the meta-analysis of sarcopenia prevalence among nonalcoholic fatty liver disease patients

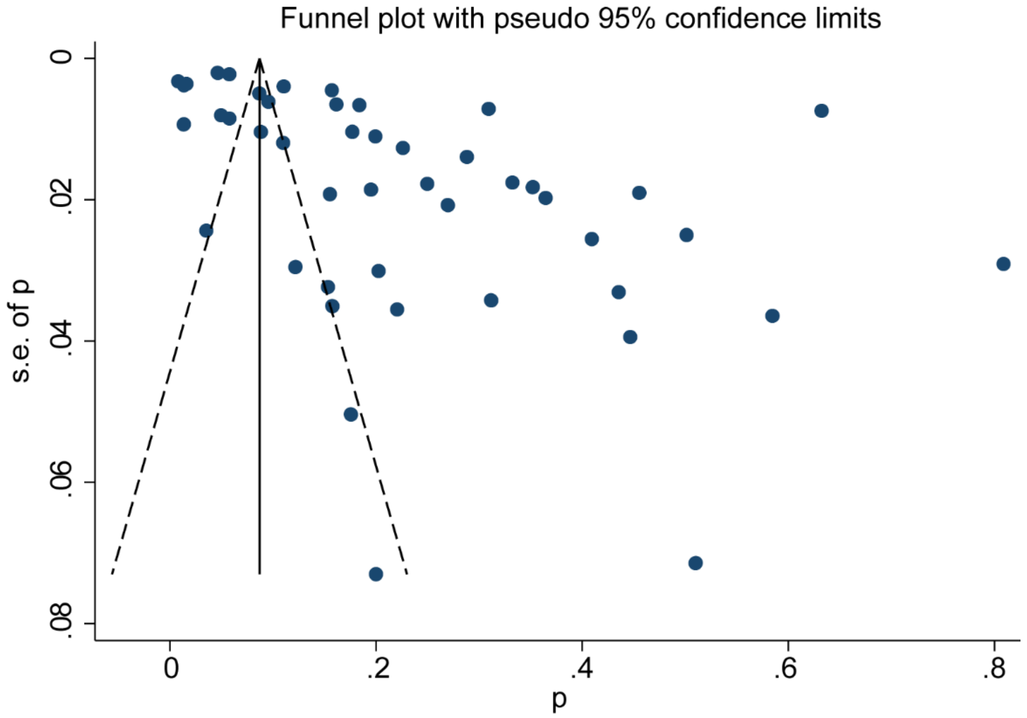

Figure S3 Funnel plot of the trim-and-fill analysis for assessing publication bias in the meta-analysis of sarcopenia prevalence among nonalcoholic fatty liver disease patients. CI, confidence interval

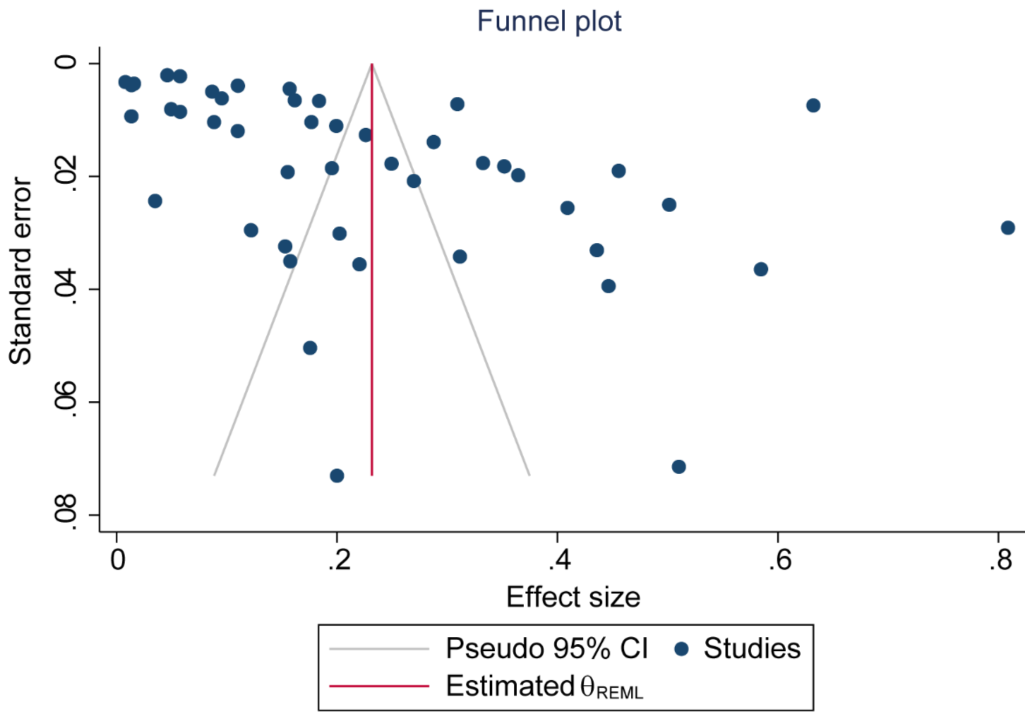

Figure S4 Sensitivity analysis for the effect of individual studies on the pooled prevalence of sarcopenia in nonalcoholic fatty liver disease. CI, confidence interval

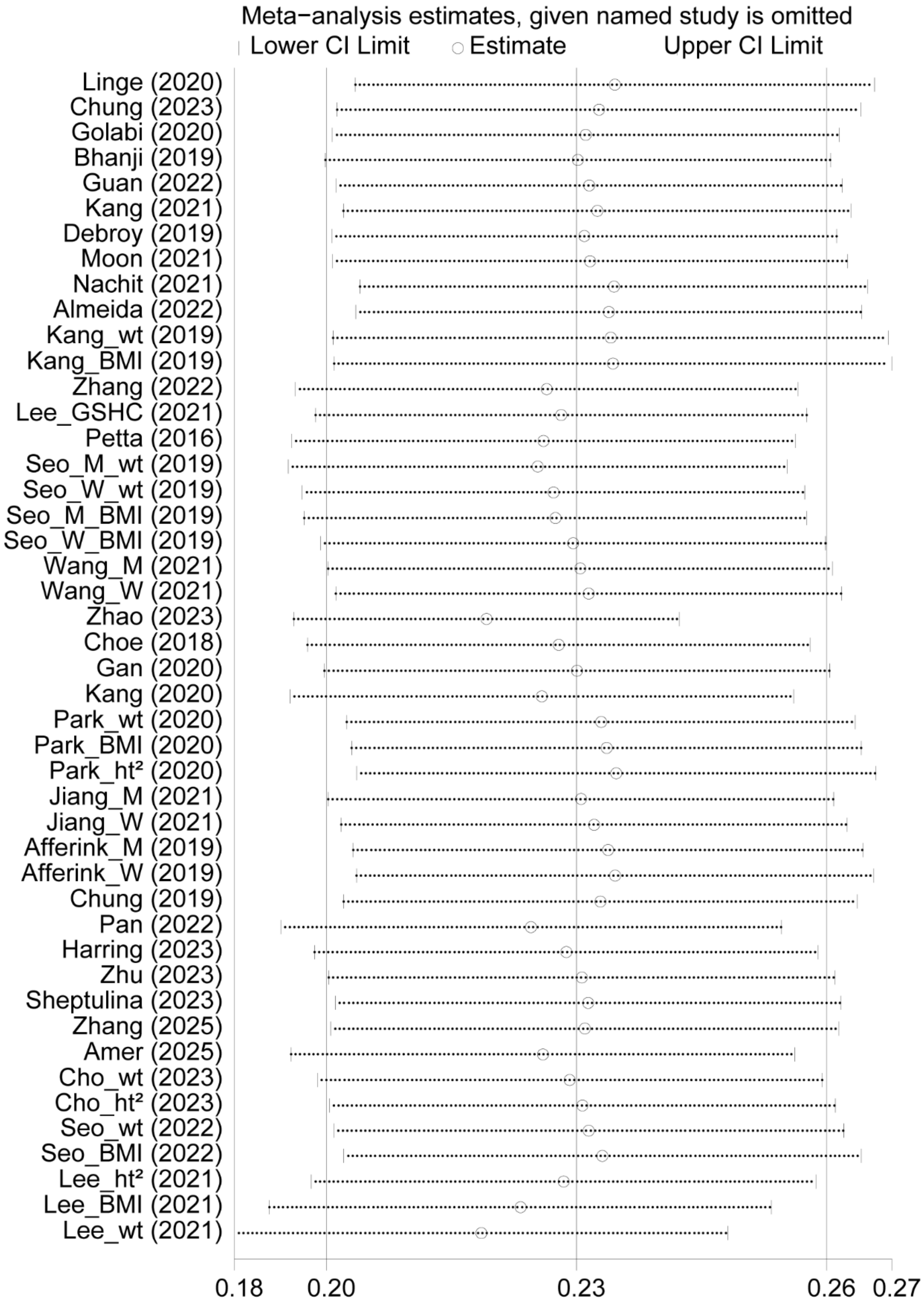

Figure S5 Funnel plot assessing publication bias in the meta-analysis of the association between sarcopenia and nonalcoholic fatty liver disease risk

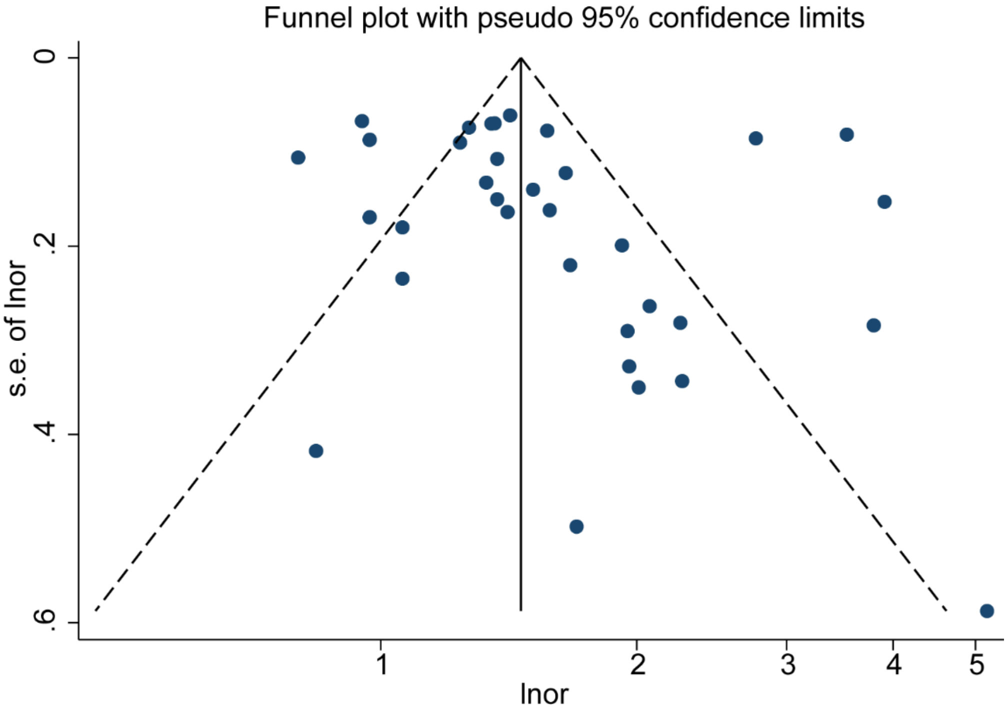

Figure S6 Sensitivity analysis of the association between sarcopenia and nonalcoholic fatty liver disease risk by omitting individual studies. CI, confidence interval

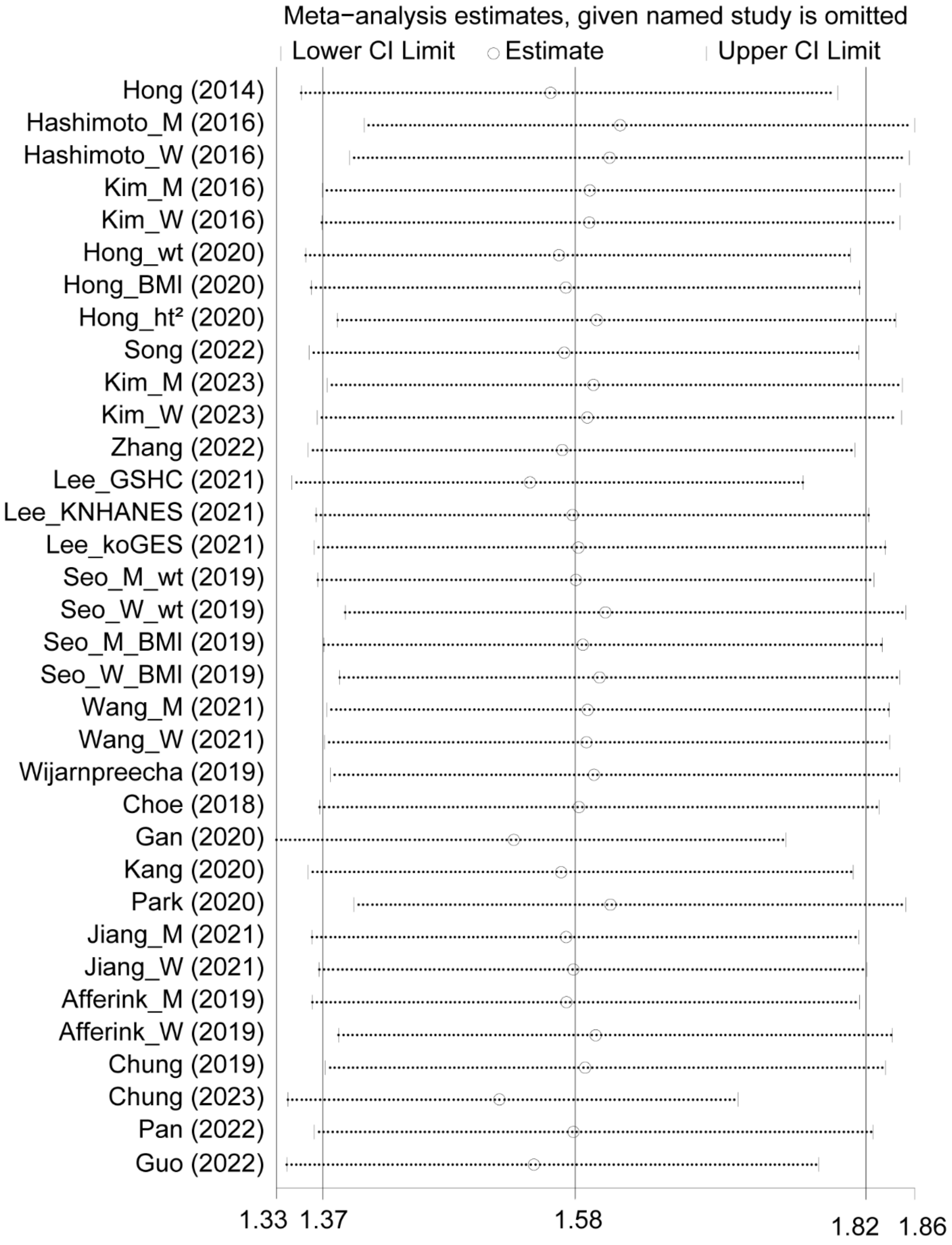

Figure S7 Funnel plot assessing publication bias in the meta-analysis of the association between sarcopenia and liver fibrosis risk

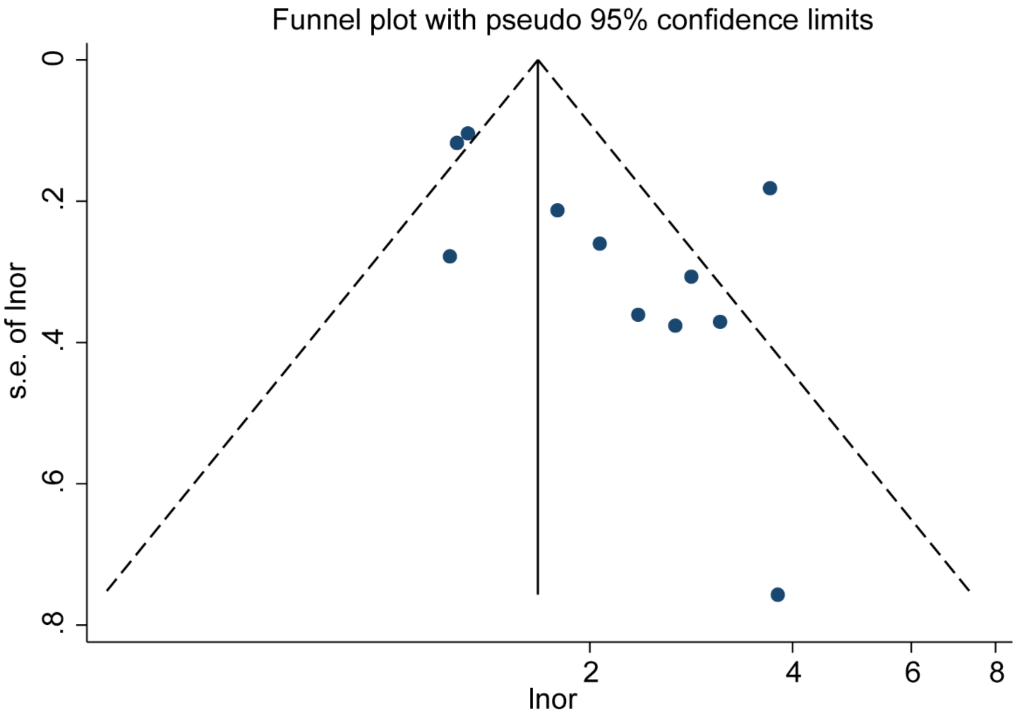

Figure S8 Funnel plot of the trim-and-fill analysis for assessing publication bias in the meta-analysis of the association between sarcopenia and liver fibrosis risk. CI, confidence interval

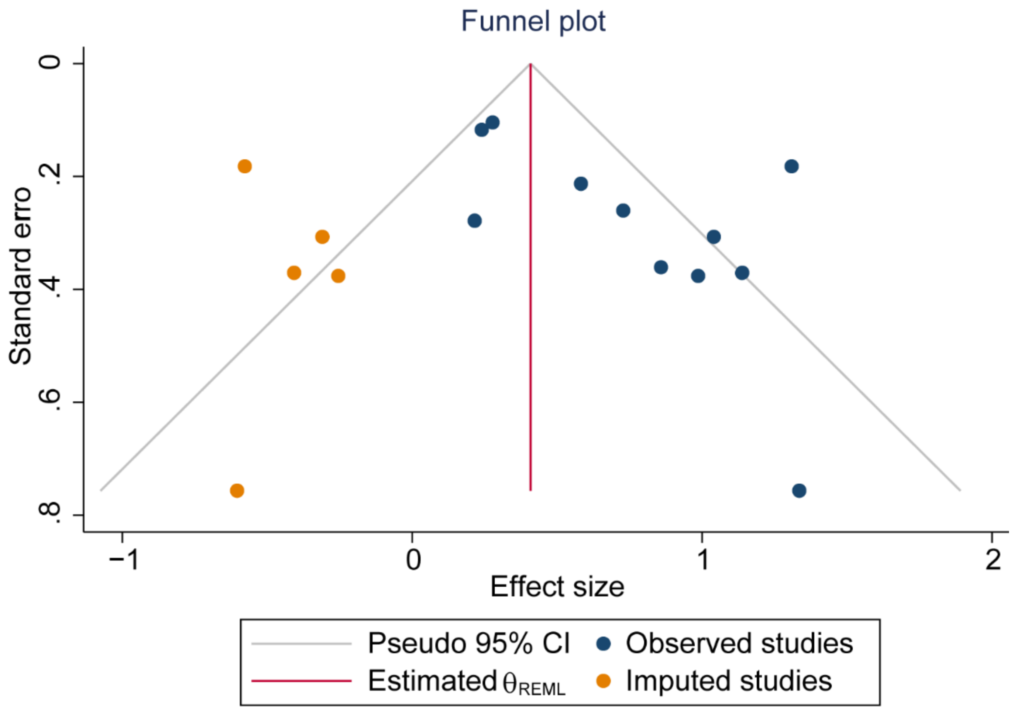

Figure S9 Sensitivity analysis of the association between sarcopenia and liver fibrosis risk by omitting individual studies. CI, confidence interval

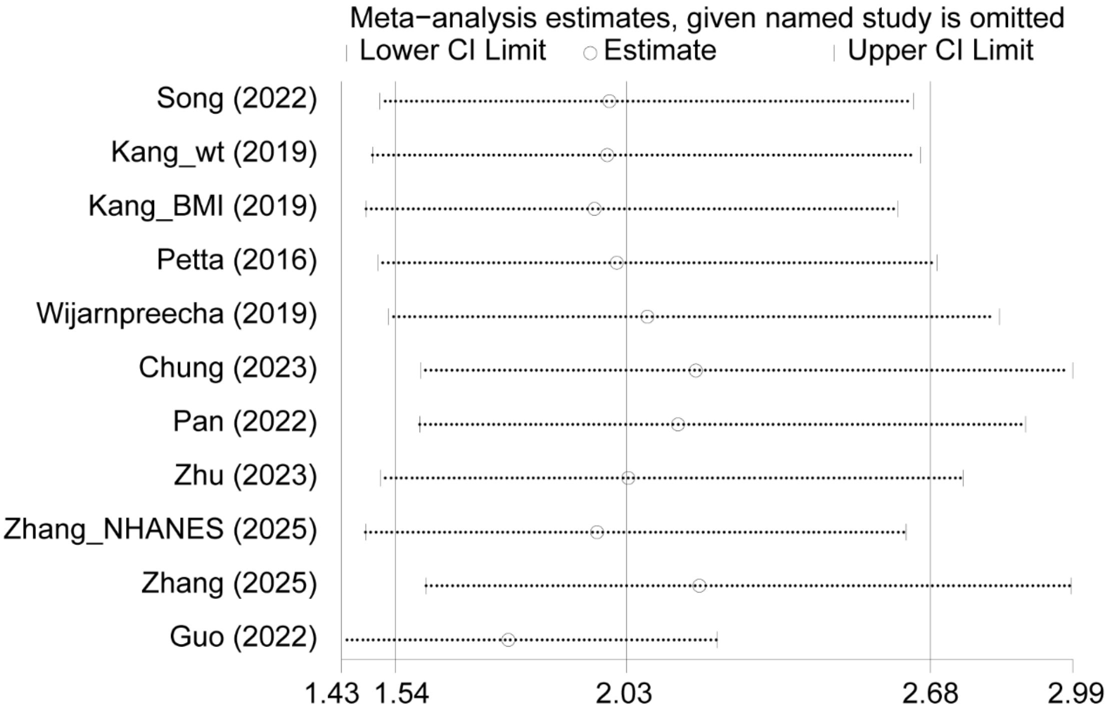

Reference

1. Kwon Y, Jeong SJ. Relative Skeletal Muscle Mass Is an Important Factor in Non-Alcoholic Fatty Liver Disease in Non-Obese Children and Adolescents. J Clin Med. 2020;9(10).doi:10.3390/jcm9103355
2. Han E, Lee YH, Kim YD, Kim BK, Park JY, Kim DY, et al. Nonalcoholic Fatty Liver Disease and Sarcopenia Are Independently Associated With Cardiovascular Risk. Am J Gastroenterol. 2020;115(4):584-95.doi:10.14309/ajg.0000000000000572
3. Yang HJ, Hong YP, Yoon TY, Ryoo JH, Choi JM, Oh CM. Independent and Synergistic Associations of Aerobic Physical Activity and Resistance Exercise with Nonalcoholic Fatty Liver Disease. Gut and Liver. 2023;17(4):600-9.doi:10.5009/gnl220345
4. Zhang S, Mak LY, Yuen MF, Seto WK. Screening strategy for non-alcoholic fatty liver disease. Clinical and Molecular Hepatology. 2023;29:S103-S22.doi:10.3350/cmh.2022.0336
5. Jung HN, Jung CH, Hwang YC. Sarcopenia in youth. Metabolism. 2023;144.doi:10.1016/j.metabol.2023.155557
6. Lee YH, Kim SU, Song K, Park JY, Kim DY, Ahn SH, et al. Sarcopenia is associated with significant liver fibrosis independently of obesity and insulin resistance in nonalcoholic fatty liver disease: Nationwide surveys (KNHANES 2008-2011). Hepatology. 2016;63(3):776-86.doi:10.1002/hep.28376
7. Pacifico L, Perla FM, Andreoli G, Grieco R, Pierimarchi P, Chiesa C. Nonalcoholic Fatty Liver Disease Is Associated With Low Skeletal Muscle Mass in Overweight/Obese Youths. Front Pediatr. 2020;8:158.doi:10.3389/fped.2020.00158
8. Moon JS, Yoon JS, Won KC, Lee HW. The role of skeletal muscle in development of nonalcoholic Fatty liver disease. Diabetes Metab J. 2013;37(4):278-85.doi:10.4093/dmj.2013.37.4.278
9. Hong J, Shin WK, Lee JW, Kim Y. Relationship Between Protein Intake and Sarcopenia in the Elderly with Nonalcoholic Fatty Liver Disease Based on the Fourth and Fifth Korea National Health and Nutrition Examination Survey. Metab Syndr Relat Disord. 2021;19(8):452-9.doi:10.1089/met.2021.0011
10. Shen H, Liangpunsakul S. Mo1555 Association Between Sarcopenia and Prevalence of Nonalcoholic Fatty Liver Disease: A Cross-Sectional Study From the Third National Health and Nutrition Examination Survey. Gastroenterology. 2016;150(4):S1143-S4.doi:10.1016/s0016-5085(16)33859-8
11. Choi YJ, Kim Sk, Kwak JJ, Park SW, Lee EJ, Huh KB. Po222 Age-Related Skeletal Muscle Loss as an Independent Predictor of Nafld Risk in Korean Women with Type 2 Diabetes. Diabetes Res Clin Pract. 2014;106:S162-S3.doi:10.1016/s0168-8227(14)70516-9
12. Kumar A, Arora A, Sharma P, Jan S, Ara I. Visceral Fat and Diabetes: Associations With Liver Fibrosis in Metabolic Dysfunction–Associated Steatotic Liver Disease. J Clin Exp Hepatol. 2025;15(1).doi:10.1016/j.jceh.2024.102378
13. Yang H, Ou F, Chang Q, Jiang J, Liu Y, Ji C, et al. Physical frailty, genetic predisposition, and the risks of severe non-alcoholic fatty liver disease and cirrhosis: a cohort study. Journal of Cachexia, Sarcopenia and Muscle. 2024;15(4):1491-500.doi:10.1002/jcsm.13506
14. Cheng Z, Hu C, Zhang Y, Zhou J, Shi J, Sun L, et al. The Different Predictive Effects of Multiple Body Fat Indexes on Metabolic Dysfunction-Associated Fatty Liver Disease. Diabetes Metab Syndr Obes. 2024;17:3875-90.doi:10.2147/DMSO.S469859
15. Wan Q, Liu X, Xu J, Zhao R, Yang S, Feng J, et al. Body Composition and Progression of Biopsy-Proven Non-Alcoholic Fatty Liver Disease in Patients With Obesity. J Cachexia Sarcopenia Muscle. 2024;15(6):2608-17.doi:10.1002/jcsm.13605
16. Bhatia SJ. Indian Journal of Gastroenterology—May–June 2023 — highlights. Indian J Gastroenterol. 2023;42(3):299-303.doi:10.1007/s12664-023-01419-8
17. Szczepanek-Parulska E, Sokołowski J, Dmowska D, Klimek J, Stasikowski T, Zdebski P, et al. Lipid profile abnormalities associated with endocrine disorders. Endokrynol Pol. 2022;73(5):863-71.doi:10.5603/EP.a2022.0059
18. Ushiro K, Matsui M, Fukuda A, Onishi S, Nishikawa T, Asai A, et al. Fatty liver index and somatic composition in subjects receiving medical health checkup. Hepatol Res. 2024.doi:10.1111/hepr.14127
19. Ushiro K, Fukuda A, Matsui M, Onishi S, Nishikawa T, Asai A, et al. Body Composition in Cases with Normal Alanine Aminotransferase Values in Medical Health Checkups. Nutrients. 2024;16(22).doi:10.3390/nu16223847
20. Tobaruela-Resola AL, Riezu-Boj JI, Milagro FI, Mogna-Pelaez P, Herrero JI, Elorz M, et al. Multipanel Approach including miRNAs, Inflammatory Markers, and Depressive Symptoms for Metabolic Dysfunction-Associated Steatotic Liver Disease Diagnosis during 2-Year Nutritional

Intervention. Nutrients. 2024;16(11).doi:10.3390/nu16111547
21. Sheptulina AF, Bryk DD, Mamutova EM, Yafarova AA, Drapkina OM. Clinical consequences of sarcopenic obesity. Part 1. Non-alcoholic fatty liver disease, type 2 diabetes mellitus, chronic kidney disease. Profilakticheskaya Meditsina. 2024;27(8):114-20.doi:10.17116/profmed202427081114
22. Onishi S, Fukuda A, Matsui M, Ushiro K, Nishikawa T, Asai A, et al. Body Composition in Patients With Metabolic Dysfunction-associated Steatotic Liver Disease: Impact of Body Surface Area. In Vivo. 2024;38(4):1917-26.doi:10.21873/invivo.13647
23. Boutari C, Stefanakis K, Simati S, Guatibonza-García V, Valenzuela-Vallejo L, Anastasiou IA, et al. Circulating total and H-specific GDF15 levels are elevated in subjects with MASLD but not in hyperlipidemic but otherwise metabolically healthy subjects with obesity. Cardiovasc Diabetol. 2024;23(1).doi:10.1186/s12933-024-02264-5
24. Polyzos SA, Vachliotis ID, Mantzoros CS. Sarcopenia, sarcopenic obesity and nonalcoholic fatty liver disease. Metabolism. 2023;147.doi:10.1016/j.metabol.2023.155676
25. Onishi S, Fukuda A, Matsui M, Ushiro K, Nishikawa T, Asai A, et al. Body Composition Analysis in Patients with Metabolic Dysfunction-Associated Fatty Liver Disease. Nutrients. 2023;15(18).doi:10.3390/nu15183878
26. Kawanaka M, Nishino K, Kawada M, Ishii K, Tanikawa T, Katsumata R, et al. Lean nonalcoholic fatty liver disease: Age-dependent differences in pathology, prognosis, and liver-related events. Hepatol Res. 2023;53(9):829-43.doi:10.1111/hepr.13911
27. Di Ciaula A, Shanmugam H, Ribeiro R, Pina A, Andrade R, Bonfrate L, et al. Liver fat accumulation more than fibrosis causes early liver dynamic dysfunction in patients with non-alcoholic fatty liver disease. Eur J Intern Med. 2023;107:52-9.doi:10.1016/j.ejim.2022.10.024
28. Shida T, Akiyama K, Oh S, Sawai A, Isobe T, Okamoto Y, et al. Skeletal muscle mass to visceral fat area ratio is an important determinant affecting hepatic conditions of non-alcoholic fatty liver disease. J Gastroenterol. 2018;53(4):535-47.doi:10.1007/s00535-017-1377-3
29. Kim LJ, Nalls MA, Eiriksdottir G, Sigurdsson S, Launer LJ, Koster A, et al. Associations of visceral and liver fat with the metabolic syndrome across the spectrum of obesity: the AGES-Reykjavik study. Obesity. 2011;19(6):1265-71.doi:
30. Onitsuka Y, Takeshima F, Ichikawa T, Kohno S, Nakao K. Estimation of visceral fat and fatty liver disease using ultrasound in patients with diabetes. Intern Med. 2014;53(6):545-53.doi:
31. Vassilatou E. Nonalcoholic fatty liver disease and polycystic ovary syndrome. World J Gastroenterol. 2014;20(26):8351-63.doi:10.3748/wjg.v20.i26.8351
32. Koch M, Borggrefe J, Schlesinger S, Barbaresko J, Groth G, Jacobs G, et al. Association of a lifestyle index with MRI-determined liver fat content in a general population study. J Epidemiol Community Health. 2015;69(8):732-7.doi:
33. Kim JY, Lee C, Oh M, Im JA, Lee JW, Chu SH, et al. Relationship between non-alcoholic fatty liver disease, metabolic syndrome and insulin resistance in Korean adults: A cross-sectional study. Clin Chim Acta. 2016;458:12-7.doi:
34. Stankevicius C, Davis RH, Huynh D, Hatzi M, Morgillo S, Day AS. Sarcopenia as a Risk Factor for Mortality in NAFLD: How Should We Diagnose It? J Dig Dis. 2024;25(11-12):645-54.doi:10.1111/1751-2980.13329
35. Mantovani A, Targher G, Zoppini G. Nonalcoholic Fatty Liver Disease and Implications for Older Adults with Diabetes. Clin Geriatr Med. 2020;36(3):527-47.doi:
36. Radmard AR, Rahmanian MS, Abrishami A, Yoonessi A, Kooraki S, Dadgostar M, et al. Assessment of Abdominal Fat Distribution in Non-Alcoholic Fatty Liver Disease by Magnetic Resonance Imaging: a Population-based Study. Arch Iran Med. 2016;19(10):693-9.doi:
37. Yen CH, Wang KT, Lee PY, Liu CC, Hsieh YC, Kuo JY, et al. Gender-differences in the associations between circulating creatine kinase, blood pressure, body mass and non-alcoholic fatty liver disease in asymptomatic asians. PLoS One. 2017;12(6).doi:10.1371/journal.pone.0179898
38. Yamamoto R, Takeshita Y, Tsujiguchi H, Kannon T, Sato T, Hosomichi K, et al. Nutrigenetic Interaction Between Apolipoprotein C3 Polymorphism and Fat Intake in People with Nonalcoholic Fatty Liver Disease. Current Developments in Nutrition. 2023;7(4).doi:10.1016/j.cdnut.2023.100051
39. Delzenne NM, Lukaski HC. Editorial: The double burn of malnutrition: the place of key nutrients revisited. Curr Opin Clin Nutr Metab Care. 2022;25(6):423-4.doi:10.1097/MCO.0000000000000875
40. Singeap AM, Stanciu C, Huiban L, Muzica CM, Cuciureanu T, Girleanu I, et al. Association between Nonalcoholic Fatty Liver Disease and Endocrinopathies: Clinical Implications. Canadian Journal of Gastroenterology and Hepatology. 2021;2021.doi:10.1155/2021/6678142
